# High visual salience of alert signals can lead to a counterintuitive increase of reaction times

**DOI:** 10.1038/s41598-024-58953-4

**Published:** 2024-04-17

**Authors:** Wolfgang Einhäuser, Christiane R. Neubert, Sabine Grimm, Alexandra Bendixen

**Affiliations:** 1 Physics of Cognition Group, Institute of Physics, Chemnitz University of Technology, Chemnitz, Germany; 2 Cognitive Systems Lab, Institute of Physics, Chemnitz University of Technology, Chemnitz, Germany; 3 BioCog – Cognitive and Biological Psychology, Institute of Psychology, Leipzig University, Leipzig, Germany

**Keywords:** Human behaviour, Attention

## Abstract

It is often assumed that rendering an alert signal more salient yields faster responses to this alert. Yet, there might be a trade-off between attracting attention and distracting from task execution. Here we tested this in four behavioral experiments with eye-tracking using an abstract alert-signal paradigm. Participants performed a visual discrimination task (primary task) while occasional alert signals occurred in the visual periphery accompanied by a congruently lateralized tone. Participants had to respond to the alert before proceeding with the primary task. When visual salience (contrast) or auditory salience (tone intensity) of the alert were increased, participants directed their gaze to the alert more quickly. This confirms that more salient alerts attract attention more efficiently. Increasing auditory salience yielded quicker responses for the alert and primary tasks, apparently confirming faster responses altogether. However, increasing visual salience did not yield similar benefits: instead, it increased the time between fixating the alert and responding, as high-salience alerts interfered with alert-task execution. Such task interference by high-salience alert-signals counteracts their more efficient attentional guidance. The design of alert signals must be adapted to a “sweet spot” that optimizes this stimulus-dependent trade-off between maximally rapid attentional orienting and minimal task interference.

## Introduction

The design of alert and warning signals faces a critical trade-off. On the one hand, the signal should direct the user’s attention towards the alert as effectively and efficiently as possible. On the other hand, the signal should not interfere with the user’s task. Well-studied aspects of such interference in applied contexts include cases where the signal itself is disturbing or harmful^1,2^, or where the signal becomes annoying to an extent that users deactivate or consistently ignore the signal^3–6^. Given that the user’s ability to react to alerts and warnings timely and adequately is crucial for the safe operation of almost any complex machinery, such as aircrafts^7^, power plants^6^, cars^8,9^, or medical equipment^10,11^, tremendous amounts of research have been dealing with the optimization of alert and warning signals—for example, on the optimal sensory modality (or modalities) in which the information should be displayed^12–16^, on the locus of presentation^17^, on the specificity of the conveyed information^18,19^, as well as on the balance between providing sufficient information and avoiding excessive mental load^20^, or on the perceived urgency in the case of warnings^21^. However, there may be an additional source of interference between alert-signal and task: an alert signal can be optimized to improve some aspect of alert processing, but this optimization may interfere with another processing stage and thus be detrimental to the overall response to the alert. In the present study, we address such a case: Can alert-signals that are optimized to attract attention towards a task-relevant item (i.e., are highly salient) interfere with task execution to such a degree that the overall performance in fact *worsens* relative to an alert that attracts attention less efficiently?

The efficiency of an alert is related to at least three components: orienting attention to the alert, responding to the alert, and resuming the primary task afterwards. To disentangle these, we devised an abstract scenario that includes a primary task and an alert-related task (alert task) that are both visual, similar in their demands, and necessitate a gaze shift to the task-related item, such that attentional orienting can be measured by eye-tracking. Using this setting, we studied the effect of low-level physical properties of visual and auditory alert signals (contrast and sound pressure level) on the three components (speed of orienting, alert response time, and time needed to resume the primary task). When it comes to such low-level features, some parts of the literature, in particular in the context of driving and traffic negotiation, show a strong focus on a signal’s “noticeability” or “salience”. For example, it has been stated that an alert signal should “*be as salient … as possible to capture the attention of individuals who might be focused on some other task*”^22^. This recommendation of high salience frequently pertains to specific features, such as size, color or contrast in the visual domain, and pulse rate or sound pressure level in the auditory domain. In a comprehensive review^23^, Laughery lists “size” and “color/contrast” as two of six key factors of warning-signal design. It is recommended that “*bigger is usually better*” though the “*important design consideration is the size of the warning relative to other displayed information*”^23^, in other words—relative size-based salience. Similarly, “*color or other forms of contrast*” (i.e., different aspects of visual salience) “*increases the noticeability*” and “*the likelihood the information will be encoded*”^23^. A representative rationale for this approach states that “*salient and powerful warning signals … direct their attention … so the unexpected hazard can be perceived faster*” and therefore “*drivers can react faster*”^24^. Similar recommendations for high saliency can be found in the literature on auditory warnings, concluding for instance that „*[t]hose who wish to obtain signals containing the highest level of perceived urgency and the shortest response time […] would employ […] the highest sound pressure level above ambient (40 dB LIN)*”^25^. It is worth noting that in these (representative) examples, faster orienting is often equated with faster perception, and faster perception in turn with faster reaction. In contrast, we here explicitly address how alert-signal properties influence these aspects differentially, and in some cases even in an opposite direction.

Complementary to the local, sensory-cognitive question how the strength of an alert signal influences different components of the response in a particular instant, there is a more global, semantic effect of stimulus strength, which has received considerable attention in the literature: the trade-off between the perceived annoyance and the perceived urgency of a warning signal. As both scale with the stimulus strength, using too strong signals to communicate urgency can become annoying and thus impair performance in the long run. In a study that covered several modalities (visual, auditory and tactile) and signal types (words, colors, pulses) in a simulated driving scenario, tactile stimuli were found to have the highest increase in perceived urgency for a given increase in annoyance^26^.^.^ In a similar setting, there was also an interaction between the semantic urgency and the sound level of a signal—warnings were most effective when they either paired a high sound level (85 dB(A)) with low-urgency semantics (“notice”) or a lower sound level (70 dB(A)) with high-urgency semantics (“danger”) as compared to the reverse combinations^27^. This suggests the existence of a sweet spot: too much urgency paired with a too strong signal (e.g. “danger” at 85 dB(A)) leads to performance that is similarly poor as too little urgency paired with too low stimulus strength. Stimulus properties may also shift subjective criteria and speed-accuracy trade-offs^28^. In the present study, we consider another trade-off moderated by low-level stimulus properties alone: the orienting of attention towards an alert, which is aided by high stimulus strength (“salience”, see below), and the distraction from the task, which is associated with such high stimulus strength. Specifically, the alert signal serves as a spatial cue to the location where the task associated with the alert shall be executed, but once the attention is oriented there, the alert signal acts as a distractor from the task. This abstract scenario mimics a real-life situation in which (for example) a message appears in the periphery that must be acknowledged before proceeding with the primary task. We use eye-tracking to explicitly measure the orienting to the alert signal, and reaction times as measures of task performance.

There is a huge body of literature in experimental psychology demonstrating distraction effects, where performance in a given task is impaired by attention-capturing stimuli or stimulus properties that are unrelated to the task^29–33^. One widely-researched phenomenon is so-called attentional capture. Attentional capture has first been demonstrated with sudden onsets: if the target appears suddenly, target detection improves, if a distractor appears suddenly, target detection deteriorates, suggesting that the sudden onset captures attention^34^. Similarly, any non-target item that is made salient by deviating from other items in a physical property (e.g., a color singleton) will slow responses by capturing attention^35^. While there is substantial debate about the details, in particular how “automatic” or unavoidable attentional capture is and to what extent it can be overridden (Ref.^36^ for a recent review), the notion that a sufficiently salient task-irrelevant item can interfere with target processing is undisputed. Our present approach is different from typical attentional-capture paradigms in one important respect. Here, the signal itself is task-relevant in that it serves as a spatial cue towards a task that has to be executed with priority. That is, the cue is task-relevant at first, but once the observer has oriented towards the target, the cue effectively becomes a distractor. We consider this relevant for applied contexts, where the role and task-relevance of stimuli or stimulus properties may often depend on the situation and change over time. Frequently, there are two or more relevant (sub)tasks (like keeping the car on track while avoiding collisions, scanning the environment and navigating towards the destination), whose relative priority intermittently needs to be reassigned (e.g. by a collision warning signal), but without all the other tasks ever becoming *entirely* irrelevant. We mimic this in our abstract task by not only requiring the participants to respond to the alert signal as quickly as possible, but also to return to the primary task immediately after executing the alert task. This allows us to study the overarching question how alert signals should be designed to optimize the trade-off between rapid orienting towards the task signaled by the alert, while minimally interfering with the execution of this task as well as of other tasks. In the present study, we specifically challenge the notion that increasing an alert signal’s salience will always lead to a more efficient execution of the alert-related task. Instead, we disentangle attentional orienting to the alert, responding to the alert and resuming the primary task. We hypothesize that increasing the salience of an alert signal too far will yield interference of the alert signal with task execution and therefore diminish the benefit of faster orienting by the cost of slower further processing.

The notion of “salience” in itself is challenging, as different branches of the literature use the term in a slightly different fashion. In the tradition of “salience-map” models^37,38^, we use the term salience strictly for stimulus-related attributes that contribute to the guidance of attention. By this definition, salience does not include current goals and selection history, which together with salience are thought to determine attentional priority^39^. Note that in Ref.^39^ the term “physical salience” is used for what we refer to as simply “salience”. All other things equal, a more salient stimulus will attract attention with higher probability and more quickly than a less salient stimulus. Here, we exploit the tight link between attention and gaze orienting^40,41^. We operationalize salience by the time it takes to orient gaze towards a stimulus (i.e. the time between the onset of the stimulus and the start of a fixation on the stimulus) measured via eye-tracking: again, all other things equal, a more salient stimulus is fixated more quickly than a less salient stimulus. This operationalization of salience allows us to compare salience across sensory modalities. We circumvent the issue of comparing physical attributes that do not share a common scale (such as contrast and sound level), but instead measure the time that is needed to fixate a stimulus and use this as operationalization of its salience. To avoid the appearance of circularity in reasoning, we emphasize that using the effect on attentional orienting for operationalization is only needed to compare the two measures across modalities, as contrast and sound pressure level are completely unrelated physical measures without a common scale (i.e. they are incommensurable). The assumption that for a pure sine tone in the absence of other sounds, auditory salience increases with sound pressure level seems trivial. Similarly, an increase of contrast is typically assumed to increase visual salience. However, as the presence of other visual stimuli, variable backgrounds and normalization might affect the definition of salience in complex scenes^42,43^, we validated for our stimuli that an increase in contrast indeed equated an increase in salience according to common salience models and metrics (Supplementary Material, part 1).

We manipulated the visual and auditory properties of alert signals and studied the impact of this manipulation on (1) orienting attention to the alert signal (its salience by the operationalization above), (2) executing the alert task, and (3) executing the primary task. We deliberately chose an abstract design that is independent of any specific application scenario. This choice includes that we deliberately did *not* manipulate several issues that are also of high relevance in practical terms, such as urgency (in our case, the alert-related task always needs to be dealt with before proceeding with the primary task), annoyance (our system cannot be ignored or switched off), competition with other alarms and warnings (there is only one alarm at a time) or surprise (the alert is comparably frequent). Rather, we focused on the issue whether an increase in efficiency to cue the user towards the alert can be detrimental for the speed of response to the alert (Experiment 1). Since we found such a detrimental effect on reaction times in Experiment 1, we asked whether this detriment can be counteracted by optimizing alert duration (Experiment 2 and 3). To foreshadow the results, we indeed found a sweet spot of alert duration for the visual signal (Experiment 2), but not for the auditory signal (Experiment 3). In Experiment 4 we therefore tested directly whether providing a high-salience visual alert signal prior to orienting and reducing its salience after orienting would indeed provide the most efficient combination in terms of minimizing reaction time to the alert. To this end, we used a gaze-contingent approach to modify the alert signal once orienting towards the alert had commenced in the current trial. That is, the alert signal was experimentally changed during the eye movement towards the alert. This allowed us to test whether it is beneficial to stop the alert once the signal has fulfilled its cueing purpose.

## Results

### Experiment 1

Experiment 1 had two aims: (i) to verify that an increase in contrast or sound intensity of an alert signal speeded up the orienting of gaze towards the alert, in line with an interpretation of contrast and sound intensity as measures of salience, (ii) to test whether thus increased salience yielded faster responses to the task associated with the alert.

**Figure 1:**
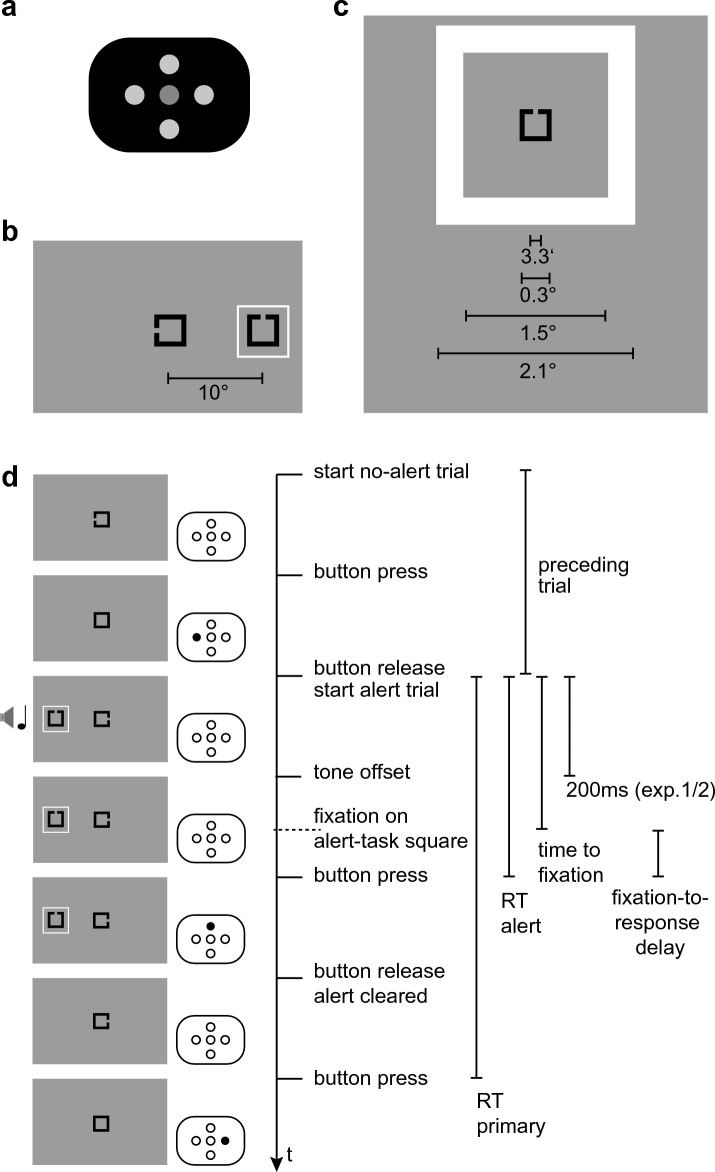
Stimuli and paradigm. (**a**) Layout of the response pad (not to scale), (**b**) layout of the screen in an alert trial (not to scale), (**c**) alert-task square and surrounding frame with relevant distances (visual angle), approximately to scale; (**d**) two trials, no-alert trial followed by alert trial, time runs from top to bottom: (i) a central square is presented with a gap (***primary task***), (ii) upon button press, the gap closes, (iii) upon button release, the next trial starts, the alert-task square, the frame and the tone are onset (***alert task***), (iv) the tone ends (in Experiment 1 and 2 after 200 ms, in Experiment 3 after a condition-dependent duration; note that the subsequent events can occur before the tone offset), (v) the participant fixates the alert square (failure to do so results in discarding the trial), (vi) the participant responds to the alert-task square, (vii) the alert-task square disappears (vii’) in Experiment 2, the frame disappears after a condition-dependent interval, independent of the conclusion of the alert task, in Experiment 3 the frame disappears after 200 ms, (viii) the participant presses the button to complete the primary task of the alert trial. The durations that are used as dependent variables throughout (time to fixation, reaction time (RT) alert task, reaction time primary task, fixation-to-response delay) are given to the right. Fixation duration is defined as the time between the start of the fixation on the alert square and its end.

To this end, we centrally presented a square that had an opening to either the left or the right. Participants (N = 20) had to indicate the side of the opening by pressing the left or right button of a 5-button response pad (Fig. 1a). On the press of the button, the opening closed and reappeared on a random side as soon as the button was released, starting the next trial. Participants first performed an initial block of 512 trials in which participants only conducted this task (hereafter: “**primary task**”). They quickly reached an approximately constant level of performance and reaction time (for details about the temporal evolution of task performance see part 4 of the Supplementary Material). Ten further blocks of 512 trials each followed. In these blocks, in 12.5% of trials (“**alert trials**”) an additional square (“**alert-task square**”) occurred in the periphery (Fig. 1b). This alert-task square was open either on the top or on the bottom. Participants had to respond to the opening of the alert-task square by pressing the top or the bottom button, before they could proceed with the central task. The size of the opening was such (about 3 arcmin, Fig. 1c) that the respective square had to be foveated for successful task execution.

Each occurrence of the alert-task square was accompanied by a surrounding frame (“**alert frame**”) and a congruently lateralized 512 Hz pure tone (“**alert tone**”). The tone had one of eight different levels of intensity (from 54 dB(A) to 89 dB(A)), and the frame had eight different levels of contrast (from 0.1 to 8.5 Weber contrast, logarithmically spaced). Alert tones lasted 200 ms; alert frames disappeared together with the alert-task square when participants responded to the alert (Fig. 1d). The lowest intensity frame or tone accompanied the different levels in the other modality, yielding 15 conditions (8 levels per modality, with the lowest levels of both being identical). Note that we carefully distinguish between the “alert *frame*”, which is varied in salience but task-irrelevant (in a sense that the task could be performed without the frame, though it modulates the speed with which the task is performed), and the alert-task *square*, which is identical for each alert trial (besides the opening) and the object the task is performed upon. That is, in our case the alert signal (the alert frame or the alert tone) is distinct from the item that is required for the response to the alert (the alert square).

Participants conducted the primary task with high accuracy, with somewhat better performance immediately after they had processed an alert (97.5% ± 2.1% of trials correct; mean ± SD) than in other (“no-alert”) trials (95.8% ± 2.3%, t(19) = 3.41, p = 0.003). Of the alert trials 11.8% ± 5.1% were excluded, as participants performed the alert task incorrectly (pressed the top button when the alert-task square was open on the bottom or vice versa), fixated the opposing side to the alert-task square first, had an error in the primary task following the alert task, or pressed the left or right button on alert occurrence first instead of the top or bottom button (Table 1). The latter case is referred to as “**alert-task intrusion**”; correspondingly, no-alert trials in which participants responded with the top or the bottom button, were classified as "**primary-task intrusion**". In the Supplementary Material, we provide details on the dependence of behavioural data on the experimental conditions (part 3) and on the serial position within a block or in the experiment (part 4) as well as a characterization in terms of sensitivity and criterion (part 5).

**Table 1:** Error types. For trials without alert (no-alert trials), we considered two types of errors: (i) *incorrect primary-task response*: the participant pressed the left button when the primary-task square was open to the right, or vice versa, (ii) *primary-task intrusion*: the participant pressed either the up or the down button (i.e., those reserved for the alert task) before pressing the left or right button. For alert trials, we considered three additional types of errors: (iii) *incorrect alert-task response*: the participant pressed the down button when the alert-task square was open to the top, or vice versa, (iv) *alert-task intrusion*: the participant pressed either the left or the right button (i.e., those reserved for the primary task) before pressing the top or bottom button, (v) *fixation error*: the first fixation that started after the onset of the alert trial was closer to the primary-task square than to the alert square (note that by definition this includes initial saccades to the wrong side). Note that the sum of the individual errors can exceed the “any error” entries, as multiple errors can occur in the same trial. Only alert trials without any error were analyzed in terms of response time.

	Error type	Experiment 1		Experiment 2		Experiment 3		Experiment 4	
		Mean (N = 20) (%)	SD (%)	Mean (N = 19) (%)	SD (%)	Mean (N = 20) (%)	SD (%)	Mean (N = 20) (%)	SD (%)
No-alert trials	Primary task intrusion	0.01	0.02	0.01	0.02	0.01	0.01	0.01	0.02
	Incorrect primary-task response	4.2	2.3	4.7	3.7	6.4	5.6	6.1	5.0
	Any error	4.2	2.3	4.7	3.7	6.4	5.6	6.2	5.0
Alert trials	Alert-task intrusion	4.7	2.8	5.7	6.1	7.2	7.3	7.1	8.3
	Incorrect alert-task response	1.1	1.0	1.7	1.5	1.4	1.2	1.3	1.5
	Fixation error	4.6	2.6	4.7	2.9	6.5	6.5	4.2	3.5
	Any error in alert task	9.7	4.0	11.3	6.7	14.2	8.6	11.6	9.5
	Primary-task intrusion	0.2	0.2	0.1	0.2	0.1	0.2	0.4	0.9
	Incorrect primary-task response	2.3	2.0	2.0	2.0	2.6	2.0	2.1	1.6
	Any error in primary task	2.5	2.1	2.1	2.0	2.7	2.0	2.5	2.2
	Any error	11.8	5.1	13.2	7.2	16.4	9.0	13.5	10.1

Since there is no reason to assume a priori that the steps in contrast and the steps in sound level are comparable, we performed separate analyses for changes in either contrast or sound level to determine whether there was any effect of these factors on the dependent variables. For analyses for which we found significant main effects, follow-up tests are reported in part 2 of the Supplementary Material.

For alert trials, we consider five different temporal measures. We defined the “**time to fixation**” as the time needed to fixate the alert-task square from the onset of the alert trial (i.e., since the simultaneous onset of primary-task square, alert square, alert frame and tone). Note that time to fixation is not directly related to a fixation duration: during the sequence of non-alert trials, fixation remains central to solve the primary task. Only with the onset of an alert trial there is an incentive to perform a saccade to the alert square surrounded by the alert frame. The fixation duration before this saccade depends on the number of non-alert trials performed, the “fixation duration” measure defined below refers to the first fixation *on* the alert square during an alert trial.

**Figure 2:**
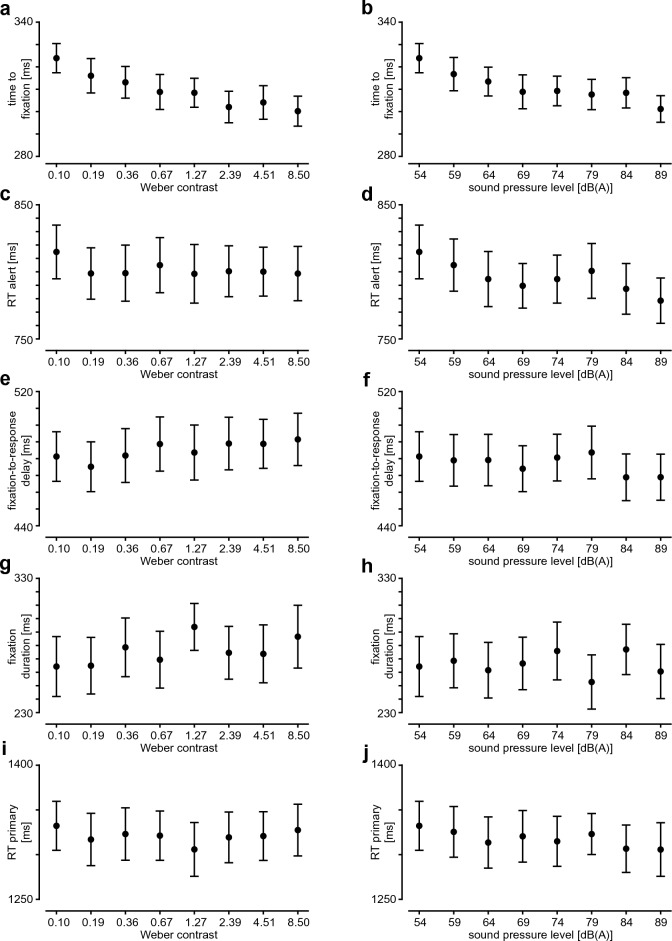
Variables of interest as a function of contrast (left) and sound pressure level (right). (**a**,**b**) time to fixation, (**c**,**d**) alert reaction time, (**e**,**f**) primary task reaction time, (**g**,**h**) fixation duration, (**i**,**j**) primary task reaction time. Results of follow-up tests in case of significant main effects are given in part 2 of the Supplementary Material.

When the contrast or the sound level was increased, the time to fixation decreased, as shown by a main effect of contrast (F(7,133) = 9.79, p < 0.001, ε = 0.51, η_G_^2^ = 0.053, η^2^ = 0.340; Fig. 2a) and sound level (F(7,133) = 7.63, p < 0.001, ε = 0.66, η_G_^2^ = 0.045, η^2^ = 0.286, Fig. 2b) on time to fixation.

We defined the **alert reaction time (“RT alert”)** as the time from trial onset to pressing the response button for the alert (top or bottom). We found no effect of contrast on this measure (F(7,133) = 1.12, p = 0.353, ε = 0.78, η_G_^2^ = 0.004, η^2^ = 0.056, Fig. 2c). RT alert did, however, decrease when sound level increased (F(7,133) = 4.89, p < 0.001, ε = 0.73, η_G_^2^ = 0.016, η^2^ = 0.205, Fig. 2d). This is a first indication that—while the time to fixation is modulated in both modalities alike—the dependence of the alert reaction time on salience is limited to the auditory domain.

The “**fixation-to-response delay”**, defined as the time from the onset of the first fixation on the alert-task square to the response to the alert-task (i.e., RT alert minus time to fixation), was not significantly affected by contrast (F(7,133) = 1.36, p = 0.246, ε = 0.73, η_G_^2^ = 0.006, η^2^ = 0.067 Fig. 2e) or sound level (F(7,133) = 1.54, p = 0.179, ε = 0.77, η_G_^2^ = 0.007, η^2^ = 0.075 Fig. 2f).

Similarly, the duration of the first fixation on the alert-task square (“**fixation duration**”) did not depend on contrast (F(7,133) = 1.64, p = 0.180, ε = 0.50, η_G_^2^ = 0.011, η^2^ = 0.080, Fig. 2g) or sound level (F(7,133) = 1.43, p = 0.218, ε = 0.73, η_G_^2^ = 0.007, η^2^ = 0.070, Fig. 2h).

The **“primary-task reaction time” (“RT primary”)** in alert trials, defined as the time from alert trial onset to pressing the response button for the primary task (left or right) after execution of the alert task, neither depended on contrast (F(7,133) = 1.00, p = 0.418, ε = 0.61, η_G_^2^ = 0.003, η^2^ = 0.050, Fig. 2i) nor on sound level (F(7,133) = 1.36, p = 0.255, ε = 0.62, η_G_^2^ = 0.005, η^2^ = 0.067, Fig. 2j).

**Figure 3:**
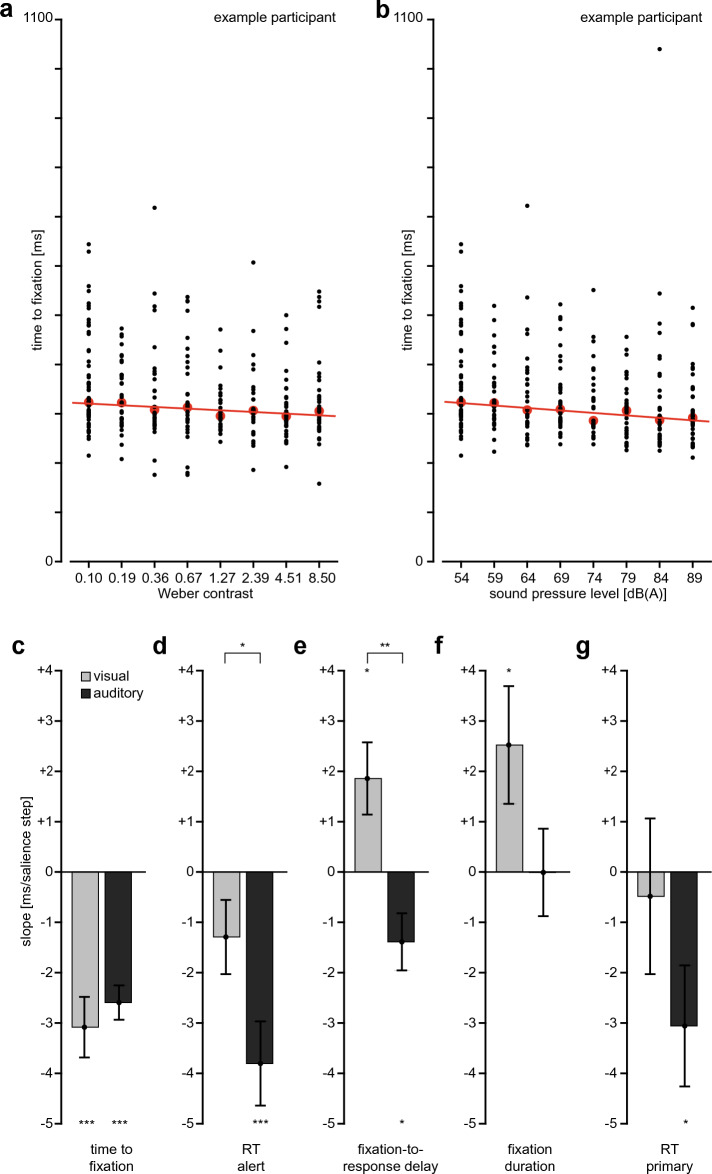
Results of Experiment 1. (**a**,**b**) “time to fixation” as function of contrast (visual salience, panel a) or sound level (auditory salience, panel b) for the same individual, black points denote individual trials, red points denote medians, line corresponds to best linear fit to median values, from which the individual slope is determined, **c-g)** mean individual slopes for time to fixation (panel c), reaction time to the alert (panel d), time from first fixation to response to the alert (panel e), fixation duration (panel f), and reaction time to the primary task in alert trials (panel g). The same vertical scale is used in all plots; errorbars denote s.e.m. across participants, significance markers at bars refer to comparisons to 0, significance markers between bars to comparison between modalities (*p < 0.05, **p < 0.01, ***p < 0.001).

For many of the variables that show a dependence on contrast and/or sound pressure level, this dependence on average seems nearly monotonic and roughly linear. To quantify the dependence of each dependent variable on contrast and sound level on an individual level, we therefore fitted linear functions to the data of each individual (Fig. 3a,b), and analyzed the resulting slopes. The slopes quantify the change in the dependent variable (one of the five duration measures) per step of visual or auditory salience.

For time to fixation, we found a negative slope for both modalities: increasing contrast at low sound levels or increasing sound level at low contrasts speeded up the time needed to fixate the alert-task square (Fig. 3c). For both modalities, the slope was negative in each individual and different from 0 at group level (visual: t(19) = 5.12, p < 0.001 ; auditory: t(19) = 7.61, p < 0.001). This speed-up of saccadic reaction times with increasing contrast and with increasing sound level is consistent with a common operationalization of “salience”: more salient stimuli yield quicker attentional orienting. Hence, the experimental manipulation of salience by modifying either contrast or sound level was successful. Importantly, the slopes were statistically indistinguishable between modalities (t(19) = 0.67, p = 0.51). Together with the fact that the lowest salience condition is identical for both modalities, the indistinguishable slopes imply that the chosen eight salience levels were comparable between the visual and the auditory modality. This justifies direct comparisons between the auditory and visual results for the remaining dependent variables.

The alert reaction time (Fig. 3d) decreased numerically with increasing visual salience (t(19) = 1.75, p = 0.10) and significantly so with increasing auditory salience (t(19) = 4.55, p < 0.001). The reaction-time benefit from increased salience was larger for auditory than for visual salience (t(19) = 2.80, p = 0.01). To analyze possible reasons for this modality difference, we analyzed the fixation-to-response delay (Fig. 3e). Indeed, this variable increased with visual salience (t(19) = 2.59, p = 0.02), while it decreased with increasing auditory salience (t(19) = 2.45, p = 0.02), with a significant difference between modalities (t(19) = 3.41, p = 0.003). Likewise fixation duration (Fig. 3f) increased with increasing visual salience (t(19) = 2.16, p = 0.04), but there was no effect for increasing auditory salience (t(19) = 0.01, p = 0.99). Both modalities tended to differ from each other (t(19) = 1.98, p = 0.06).

The primary-task reaction time (Fig. 3g) decreased with an increase in auditory salience (t(19) = 2.54, p = 0.02). An effect of visual salience was not observed (t(19) = 0.31, p = 0.76), and we did not observe a difference between the modalities (t(19) = 1.52, p = 0.14). Hence, while auditory and visual salience had the same effect with respect to speeding-up orienting to the alert-task square (Fig. 3c), increasing auditory salience speeded up the behavioral response more than increasing visual salience (Fig. 3d,g), mostly as a consequence of a difference in the time from fixation to response (Fig. 3e).

In sum, visual and auditory salience increases had the same beneficial effect on attentional orienting, but quite different effects on subsequent processing: while reaction times benefitted from high auditory salience, high visual salience prolonged fixation durations and slowed down responses, counteracting or even cancelling the orienting benefit.

The results of Experiment 1 raise two new questions: (i) While contrast and sound pressure level speed up orienting similarly (i.e., the levels used in the present experiment are about matched in salience), they are still physically different and thus in principle incommensurable. Ideally, one would like to assess the effects of a stimulus property that can be varied in both modalities on the same scale to address the question as to where the differential effects on subsequent processing originate. (ii) If the alert frame is indeed distracting and therefore hampering the reaction to the alert at high salience levels, removing it after a certain time should alleviate this effect, especially if it is removed after the orienting response has already been initiated. Again, this would provide insights into the origin of the effects on alert- and primary-task-related costs and benefits. To address both questions, we varied alert duration, which has previously been described to affect response times^44^, in two additional experiments. In Experiment 2, we manipulated the duration of the alert frame, in Experiment 3 the duration of the alert tone.

### Experiment 2

In Experiment 2, we varied the duration of the alert frame. We tested whether shortening or prolonging the alert signal in time had similar effects as decreasing or increasing its contrast. Unlike in Experiment 1, where the alert frame disappeared together with the alert-task square upon button release, in Experiment 2, the alert frame had a fixed presentation duration. To cover a wide range of durations, we chose eight different levels ranging from 25 to 800 ms. Moreover, by using two contrast levels (0.10 and 2.39 Weber contrast), we tested whether the signal’s duration interacted with the signal’s contrast. The contrast levels were chosen because around a Weber contrast of 2.39, RT alert leveled off in Experiment 1 (Fig. 2c) and the fixation-to-response delay increased when contrast increased beyond this level (Fig. 2e). To match the visual conditions of Experiment 1 closely, the congruently lateralized tone was presented at 54 dB(A) in all trials. In all other aspects, Experiment 2 was identical to Experiment 1 (except for using N = 19, as one participant had to be excluded because of low performance).

Participants performed the primary task correctly and without intrusions in 95.3% ± 3.7% of no-alert trials, and in 97.9% ± 2.0% of alert trials (Table 1; difference: t(18) = 3.20, p = 0.005).

**Figure 4:**
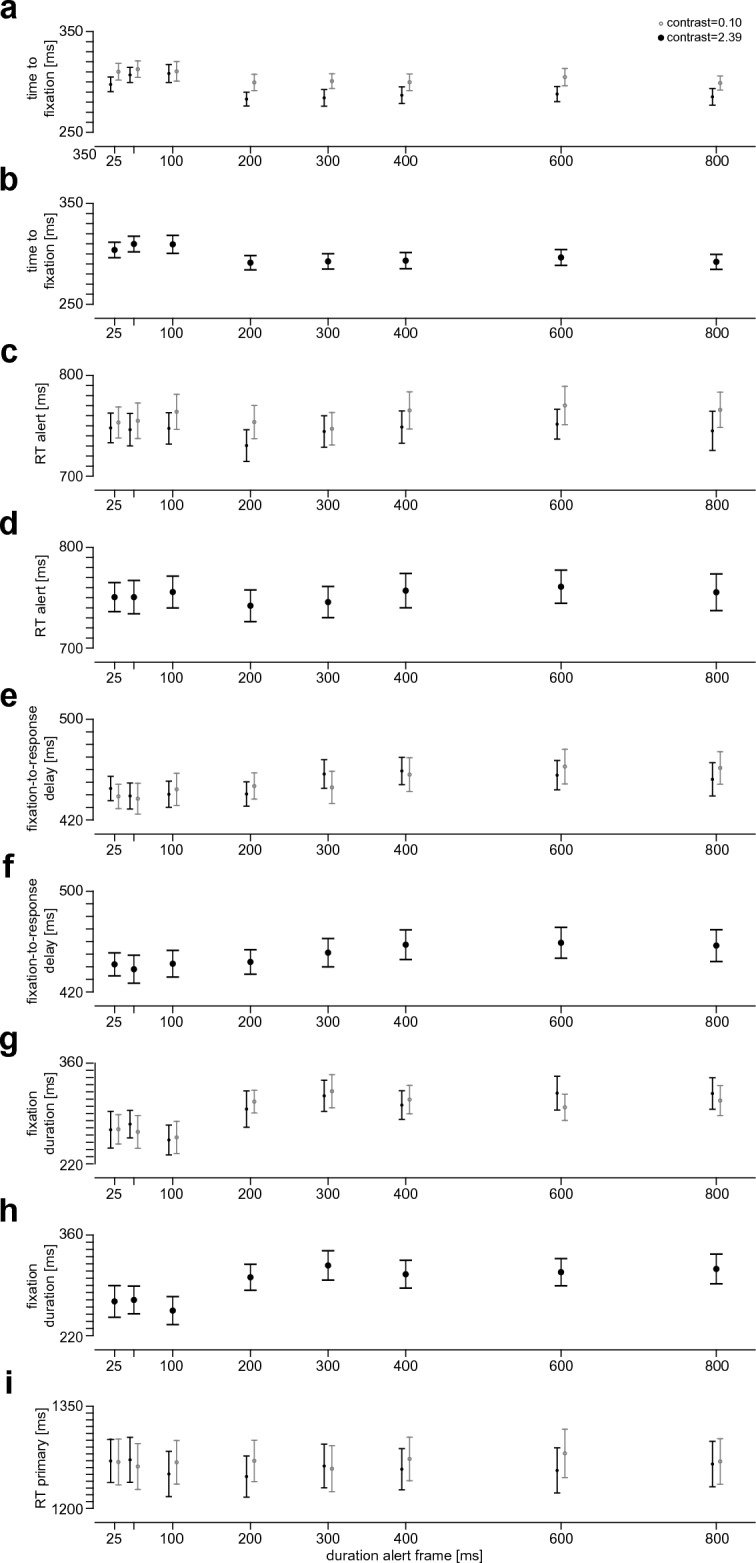
Results of Experiment 2. (**a**) Time to first fixation on the alert square as function of alert-frame duration for the two different contrast levels, mean and s.e.m. across participants; (**b**) Data of panel a averaged across contrast levels; mean and s.e.m. across participants; (**c**) reaction time to the alert as function of alert-frame duration, notation as in panel a; (**d**) data of panel c averaged across contrast levels; notation as in panel b; (**e**) time from first fixation on the alert square to the response to the alert as function of alert-frame duration, notation as in panel a; (**f**) data of panel e averaged across contrast levels; notation as in panel b; (**g**) fixation duration as function of alert-frame duration, notation as in panel a (**h**) data of panel g averaged across contrast levels; notation as in panel b; (**i**) reaction time to the primary task in alert trials as function of alert-frame duration, notation as in panel a.

The time to fixation (Fig. 4a) depended on visual salience (contrast of the frame; F(1,18) = 46.83, p < 0.001, η_G_^2^ = 0.031, η_p_^2^ = 0.722) and on the duration for which the alert frame was visible (F(7,126) = 22.96, p < 0.001, ε = 0.60, η_G_^2^ = 0.045, η_p_^2^ = 0.561). Since we observed no significant interaction between these two factors (F(7,126) = 2.30, p = 0.062, ε = 0.61, η_G_^2^ = 0.005, η_p_^2^ = 0.113), we collapsed the data across the two salience levels for further analysis (Fig. 4b). Pairwise comparisons between different alert-frame durations suggested that they fall into two groups with a step between 100 and 200 ms: for each alert-frame duration of 200 ms and above, the time to fixation was significantly shorter than for each alert-frame duration of 100 ms and below (all p < 0.006) with only one pair not withstanding Bonferroni-Holm correction (Supplementary Table S2a). None of the other pairs shows a significant difference after this correction (Supplementary Table S2a). That is, in general, alert-frame durations of 200 ms and above yielding quicker attentional orienting than alert-frame durations of 100 ms and below.

The alert reaction time (Fig. 4c) depended on salience (F(1,18) = 21.85, p < 0.001, η_G_^2^ = 0.01, η_p_^2^ = 0.548) and alert-frame duration (F(7,126) = 2.60, p = 0.03, ε = 0.72, η_G_^2^ = 0.007, η_p_^2^ = 0.126), with no interaction between those factors (F(7,126) = 0.96, p = 0.45, ε = 0.76, η_G_^2^ = 0.002, η_p_^2^ = 0.051). Numerically, a minimum RT was reached at 200 ms alert-frame duration, after which the reaction time continued to increase up to an alert-frame duration of 600 ms (Fig. 4d). Similar as for contrast in Experiment 1, the quicker orienting for longer alert-frame durations therefore did not result in a continuous speed-up of the responses. Instead, there seems to be an optimal alert-frame duration, beyond which reaction time is rather hampered than helped. Consistent with this observation, the fixation-to-response delay (Fig. 4e) increased with stimulus duration (F(7,126) = 5.90, p < 0.001, ε = 0.69, η_G_^2^ = 0.024, η_p_^2^ = 0.247). Neither a main effect of salience (F(1,18) = 0.03, p = 0.86, η_G_^2^ < 0.0001, η_p_^2^ = 0.002) nor an interaction of salience and duration (F(7,126) = 1.08, p = 0.37, ε = 0.68, η_G_^2^ = 0.004, η_p_^2^ = 0.057) was observed for the fixation-to-response delay. Similarly, fixation duration (Fig. 4f) depended on alert-frame duration (F(7,126) = 6.69, p < 0.001, ε = 0.70, η_G_^2^ = 0.058, η_p_^2^ = 0.271), while no main effect of salience (F(1,18) = 0.07, p = 0.80, η_G_^2^ = 0.00006, η_p_^2^ = 0.004) and no interaction (F(7,126) = 0.42, p = 0.78, ε = 0.53, η_G_^2^ = 0.003, η_p_^2^ = 0.023) was found. Post-hoc analysis showed that the difference in fixation duration arises between alert-frame durations of 100 ms and 300 ms, with fixation durations being significantly shorter for alert-frame durations of 100 ms and below than for alert-frame durations at and above 300 ms (Fig. 4g). Consistent with the alert reaction times, the primary-task reaction time (Fig. 4h) depended on salience (F(1,18) = 5.09, p = 0.004, η_G_^2^ = 0.001, η_p_^2^ = 0.220), but neither a main effect of alert-frame duration (F(7,126) = 0.72, p = 0.60, ε = 0.67, η_G_^2^ = 0.001, η_p_^2^ = 0.039) nor an interaction (F(7,126) = 1.35, p = 0.25, ε = 0.68, η_G_^2^ = 0.002, η_p_^2^ = 0.070) was observed.

In sum, an increase in alert-frame duration had an analogous effect to an increase in contrast in Experiment 1: longer durations (here beyond 200 ms) resulted in quicker orienting to the alert-task square, but also prolonged the time from fixating the alert to the response. Consequently, extending the alert duration beyond a certain “sweet spot” can harm the efficiency of the response to the alert. Since the dependence on alert-frame duration was step-like for time to fixation and clearly non-monotonic for the other variables, a linear fit akin to Experiment 1 would not be an appropriate model for the dependencies observed in Experiment 2. The main effects of salience observed in Experiment 2 are largely consistent with the data of Experiment 1: In Experiment 2, we used 0.10 and 2.39 as the two levels of Weber contrast and found main effects of salience for time to fixation, RT alert and RT primary. For time to fixation and RT alert, we had also found significant differences between these two contrast levels (0.10 and 2.39) in Experiment 1 (Supplementary Table S1). The only difference between experiments was seen for RT primary, which depended on visual salience in Experiment 2, but not in Experiment 1 (though, if the two contrast levels used in Experiment 2 were considered in isolation in Experiment 1, there also would have been a significant effect, t(19) = 3.30, p = 0.004), again in line with the result of Experiment 2).

Together, the results of Experiment 1 on visual contrast and of Experiment 2 on visual contrast and duration leave room for two interpretations: (i) alert duration could act like visual salience (contrast)—there is an optimum after which a further increase becomes harmful to task execution—in general, that is, irrespective of the sensory modality. Or, (ii), this behavior could be specific to vision (i.e., the modality used for the alert task), irrespective of whether contrast or duration is tested. By varying the alert-tone duration, we directly test these alternatives in Experiment 3.

### Experiment 3

Whereas the duration and contrast of the visual alert (the alert frame) were varied in Experiment 2, in Experiment 3 we varied the duration and salience of the auditory alert (the alert tone). Since duration is commensurate across modalities, we varied the duration of the alert tone using the same durations (eight levels ranging from 25 to 800 ms) used for the frame in Experiment 2. Note that a priori, auditory and visual alerts might have distinct optimal time scales (cf. Refs.^3,45^). This justifies the use of a broad range of durations to use the same values for both modalities, while at the same time safely covering the potentially relevant range for either modality. In analogy to Experiment 2, two distinct sound-pressure levels were used, 54 dB(A) and 79 dB(A), corresponding to the first and sixth level of Experiment 1 (as the contrasts in Experiment 2 also matched the first and sixth level of Experiment 1), and the alert frame was used at its lowest contrast (0.10) and with a fixed duration of 200 ms throughout.

Participants (N = 20) performed the primary task correctly and without intrusions in 93.6% ± 5.6% of no-alert trials, and in 97.3% ± 2.0% of alert trials (Table 1; difference: t(19) = 3.63, p = 0.002).

**Figure 5:**
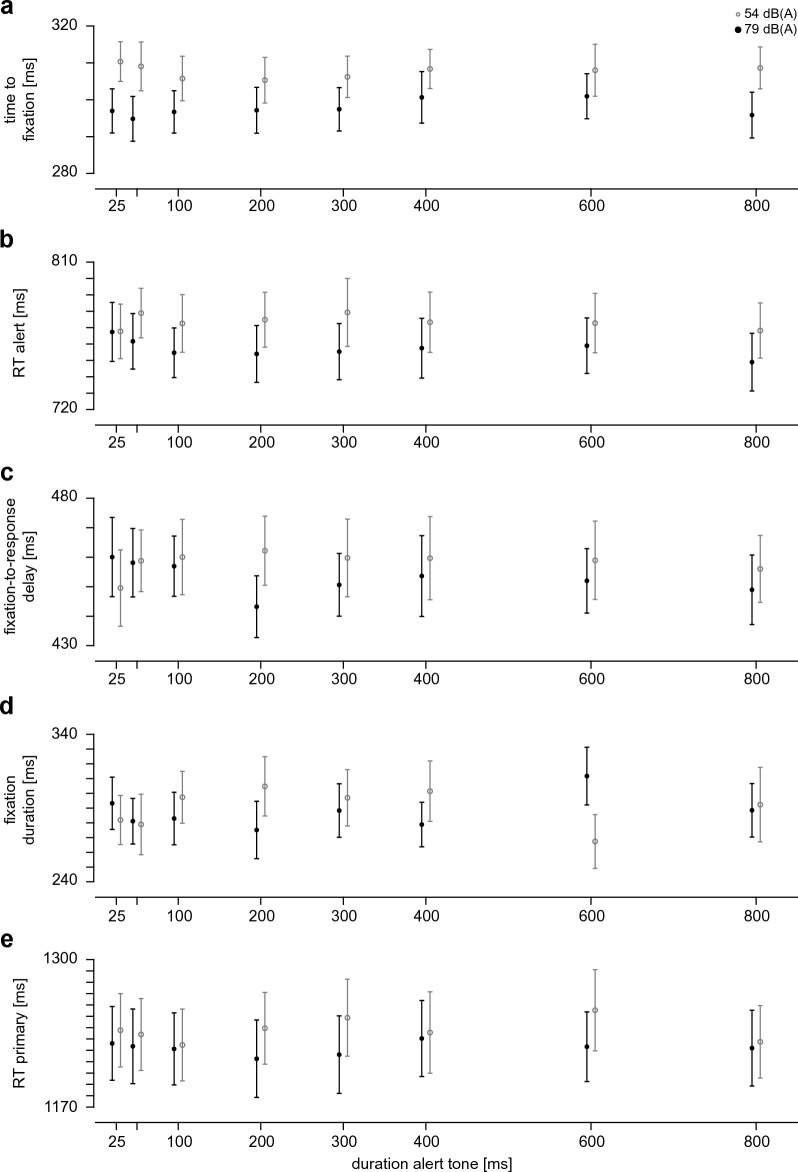
Results of Experiment 3. (**a**–**e**) Dependent variables as function of alert-tone duration, sound level denoted by grayscale as indicated in panel a; mean and s.e.m. across participants. (**a**) time to fixation on alert-task square, (**b**) reaction time to the alert, (**c**) time from fixating the alert-task square to responding to the alert task, (**d**) duration of first fixation on the alert-task square, (**e**) reaction time to the primary task in alert trials.

The time to fixation (Fig. 5a) depended on auditory salience (sound level; F(1,19) = 49.93, p < 0.001, η_G_^2^ = 0.035, η_p_^2^ = 0.724), but neither an effect of alert-sound duration (F(7,133) = 0.75, p = 0.575, ε = 0.63, η_G_^2^ = 0.002, η_p_^2^ = 0.038) nor an interaction between these factors was observed (F(7,133) = 0.82, p = 0.523, ε = 0.60, η_G_^2^ = 0.003, η_p_^2^ = 0.041). The same pattern of results applied to the alert reaction time (Fig. 5b; main effect of salience: F(1,19) = 46.28, p < 0.001, η_G_^2^ = 0.011, η_p_^2^ = 0.709; main effect of duration: F(7,133) = 0.72, p = 0.576, ε = 0.56, η_G_^2^ = 0.002, η_p_^2^ = 0.037; interaction: F(7,133) = 1.02, p = 0.405, ε = 0.57, η_G_^2^ = 0.002, η_p_^2^ = 0.051), the fixation-to-response delay (Fig. 5c; main effect of salience: F(1,19) = 5.92, p = 0.025, η_G_^2^ = 0.002, η_p_^2^ = 0.237; main effect of duration: F(7,133) = 0.44, p = 0.808, ε = 0.67, η_G_^2^ = 0.002, η_p_^2^ = 0.023; interaction: F(7,133) = 1.88, p = 0.109, ε = 0.68, η_G_^2^ = 0.005, η_p_^2^ = 0.090) and to the primary-task reaction time (Fig. 5e; main effect of salience: F(1,19) = 65.25, p < 0.001, η_G_^2^ = 0.003, η_p_^2^ = 0.774; main effect of duration: F(7,133) = 1.25, p = 0.292, ε = 0.76, η_G_^2^ = 0.001, η_p_^2^ = 0.062; interaction: F(7,133) = 1.45, p = 0.214, ε = 0.70, η_G_^2^ = 0.002, η_p_^2^ = 0.071). For fixation durations (Fig. 5d), no main effect of either factor (salience: F(1,19) = 0.27, p = 0.612, η_G_^2^ = 0.0003, η_p_^2^ = 0.014 duration: F(7,133) = 0.27, p = 0.897 ε = 0.56, η_G_^2^ = 0.002, η_p_^2^ = 0.014 nor an interaction (F(7,133) = 0.98, p = 0.399 ε = 0.36, η_G_^2^ = 0.017, η_p_^2^ = 0.049) was observed.

Regarding auditory salience, the results of Experiment 3 are largely in line with those of Experiment 1. We found main effects of sound level in Experiment 3 for the same dependent variables that showed a significant linear dependence on sound level—i.e. a slope significantly smaller than 0—in Experiment 1 (time to fixation, alert reaction time, fixation-to-response delay, primary task RT). Note that only two of these four dependent variables showed a significant main effect of sound level in the ANOVAs of Experiment 1. Given that in Experiment 3 we have only two levels, the same total number of trials and little interaction with duration, and given that we have chosen salience levels that are far apart, it is not surprising that the ANOVA in Experiment 3 provides more robust results than in Experiment 1, where the dense sampling of salience levels makes the slope-based analysis more robust if there is indeed the linear dependence we observe. Importantly, we found no effects of alert-tone duration, which is markedly different from the effect of alert-frame duration observed in Experiment 2. Hence, the effects of alert duration seem to be largely specific to the sensory modality used for the alert task.

### Experiment 4

Based on the results of Experiments 1 through 3, it seems plausible that higher salience of the alert signal (the alert frame or tone) aids attentional orienting, but once the alert square is fixated, higher visual salience interferes with task execution. To test this hypothesis directly, in Experiment 4, we used a gaze-contingent paradigm. This allowed us to independently vary the contrast of the alert frame before the gaze was shifted towards the alert-task square (before saccade) and after the gaze was shifted (after saccade). Two levels of contrast (low: 0.10 and high: 2.39) were used. This provided a 2 × 2 design on visual salience, separating the factors visual salience (contrast) *before* the saccade (low or high) and visual salience *after* the saccade (low or high). As an additional factor, we paired these visual stimuli with 2 levels of auditory salience (54 dB(A), 79 dB(A)) in a total 2 × 2 × 2 design. Since the alert-tone duration had little effect in Experiment 3, we chose to reduce the tone duration to 50 ms, such that the tone would be finished prior to the execution of the saccade. Based on our interpretation of the findings of Experiment 1 through 3, we hypothesized that contrast before the saccade influences the *orienting* to the alert, while contrast after the saccade influences the *response* to the alert. Specifically, with respect to the contrast before the saccade, we expected that high contrasts yield a shorter time to fixation and *faster* reaction times than low contrasts. In turn, we expected that a high contrast after the saccade will lead to *slower* reaction times and a larger fixation-to-response delay than a low contrast after the saccade.

Participants (N = 20) performed the primary task correctly and without intrusions in 93.8% ± 5.0% of no-alert trials, and in 97.5% ± 2.2% of alert trials (difference: t(19) = 3.98, p = 0.001).

**Figure 6:**
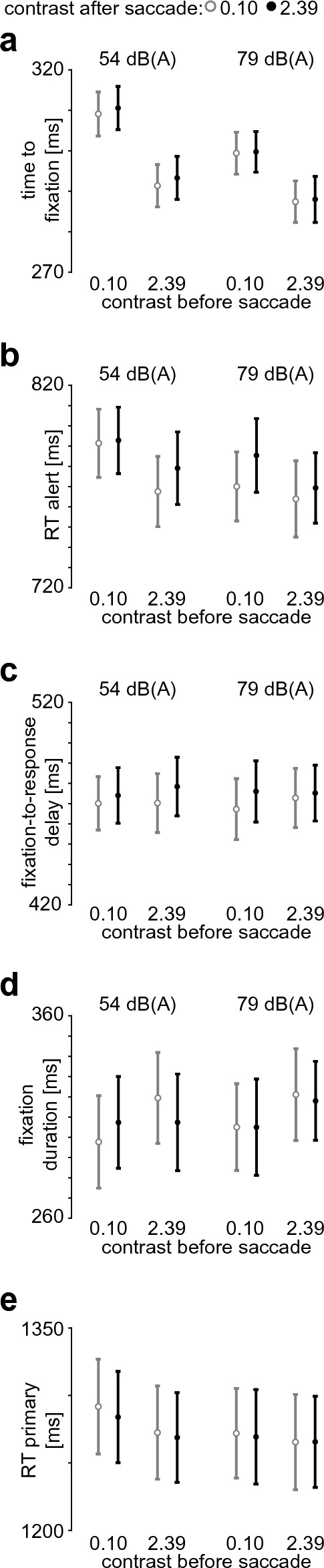
Results of Experiment 4. (**a**-**e**) Dependent variables as function of sound level, contrast before the saccade and contrast after the saccade (see legend on top); (**a**) time to fixation on alert-task square, (**b**) reaction time to the alert task, (**c**) time from fixating the alert-task square to responding to the alert task, (**d**) duration of first fixation on the alert-task square, (**e**) reaction time to the primary task in alert trials.

For time to fixation (Fig. 6a), there was a main effect of sound level (F(1,19) = 31.17, p < 0.001, η_G_^2^ = 0.025, η_p_^2^ = 0.621) and of contrast before the saccade (F(1,19) = 137.36, p < 0.001, η_G_^2^ = 0.092, η_p_^2^ = 0.878). Since the level of contrast after the saccade by definition only becomes evident after the saccade has been executed, it cannot influence the time to fixation. Hence, it is a sanity check that there is no main effect of contrast after the saccade, which was indeed the case (F(1,19) = 0.52, p = 0.48, η_G_^2^ = 0.0005, η_p_^2^ = 0.026). We found a significant two-way interaction for sound level and contrast before the saccade (F(1,19) = 6.44, p = 0.02, η_G_^2^ = 0.004, η_p_^2^ = 0.253), but no other significant two-way or three-way interactions (all F(1,19) < 0.36, all p > 0.55, all η_G_^2^ < 0.0002, all η_p_^2^ < 0.019).

For the alert reaction time (Fig. 6b), we found a three-way interaction between sound level, the contrast before, and the contrast after the saccade (F(1,19) = 4.52, p = 0.047, η_G_^2^ = 0.001, η_p_^2^ = 0.192) as well as main effects of sound level (F(1,19) = 9.64, p = 0.006, η_G_^2^ = 0.005, η_p_^2^ = 0.337), contrast before the saccade (F(1,19) = 31.72, p < 0.001, η_G_^2^ = 0.009, η_p_^2^ = 0.625) and contrast after saccade (F(1,19) = 12.96, p = 0.002, η_G_^2^ = 0.003, η_p_^2^ = 0.406). To assess the three-way interaction, we analyzed the effects of the contrasts separately for the two sound levels. For the low-salience tone (54 dB(A)), we found a main effect of contrast before the saccade (F(1,19) = 21.66, p < 0.001, η_G_^2^ = 0.016, η_p_^2^ = 0.533) with faster responses for the higher contrast, and a trend towards a main effect of the contrast after the saccade (F(1,19) = 4.28, p = 0.052, η_G_^2^ = 0.002, η_p_^2^ = 0.184) with slower responses for higher contrasts. We did not observe an interaction (F(1,19) = 2.87, p = 0.11, η_G_^2^ = 0.001, η_p_^2^ = 0.131). Similarly, for the high-salience tone (79 dB(A)), we found a main effect of contrast before the saccade (F(1,19) = 10.06, p = 0.005, η_G_^2^ = 0.005, η_p_^2^ = 0.346), with faster responses for higher contrasts. There was also a main effect for contrast after the saccade (F(1,19) = 8.84, p = 0.008, η_G_^2^ = 0.004, η_p_^2^ = 0.318) with slower responses for higher contrasts. There was no interaction for contrast before and after saccade (F(1,19) = 1.86, p = 0.19, η_G_^2^ = 0.001, η_p_^2^ = 0.089).  In sum, high visual salience *before* the saccade yields *faster* responses to the alert task while high visual salience *after* the saccade yields *slower* responses.

For the time between fixating the alert-task square and the reaction to the alert (Fig. 6c), we found a main effect of the contrast after the saccade (F(1,19) = 8.70, p = 0.008, η_G_^2^ = 0.002, η_p_^2^ = 0.314), where higher contrast increased this delay. We did not observe any other main effects, two-way or three-way interactions (all F(1,19) < 1.96, all p > 0.18, all η_G_^2^ < 0.0005, all η_p_^2^ < 0.093). This supports the notion that high salience after the saccade increases the time from orienting to the alert to responding.

For fixation durations (Fig. 6d), a significant main effect of contrast before the saccade was observed (F(1,19) = 6.26, p = 0.02, η_G_^2^ = 0.004, η_p_^2^ = 0.248), but no other significant main effects or interactions (all F(1,19) < 0.76, all p > 0.39, all η_G_^2^ < 0.001, all η_p_^2^ < 0.039).

For the primary-task reaction time (Fig. 6e), we found a main effect of sound level (F(1,19) = 9.89, p = 0.005, η_G_^2^ = 0.001, η_p_^2^ = 0.342), contrast before the saccade (F(1,19) = 6.95, p = 0.02, η_G_^2^ = 0.001, η_p_^2^ = 0.268), and an interaction between these two factors (F(1,19) = 5.41, p = 0.03, η_G_^2^ < 0.0004, η_p_^2^ = 0.222). Higher sound level increased the speed of the primary task reaction, as did higher contrast before the saccade, but predominantly at low sound levels. No further main effects or interactions were observed (all F(1,19) < 0.87, all p > 0.36, all η_G_^2^ < 0.0002, all η_p_^2^ < 0.044). That is, high visual or auditory salience at the start of the trial yields faster primary-task responses, and this speed-up is more pronounced if the salience in the other modality is low.

In sum, Experiment 4 underlines that high salience in either modality before the saccade can yield overall response benefits, but high visual saliency after orienting towards the alert impairs processing.

## Discussion

In a series of four experiments, we investigated how visual and auditory properties of alert signals affect human responses to them, and specifically whether different facets of alert and task responses would be differentially affected by alert-signal properties. We disentangled effects on attentional orienting (operationalized as *time to fixation*), on the execution of the alert task (*RT alert*), and on the execution of the primary task (*RT primary*). We found that the levels chosen for auditory and visual alerts (sound level and contrast) had quantitatively indistinguishable effects on the time needed to fixate the alert signal. Taking time to fixation as a proxy of attentional orienting, we infer that visual and auditory levels were matched for their salience. Importantly, visual and auditory salience had distinct effects on the efficiency of task execution. While the increase in auditory salience translated into a reaction time benefit, for visual salience the orienting benefit was counteracted by a prolonged time between orienting to the alert and executing the behavioral response. Similarly, an increase in the duration of the visual alert yielded a quicker orienting response, but too long of a visual alert again reduced the efficiency in responding to the alert. These findings show that increasing the salience of an alert signal does not necessarily induce a more efficient response.

Our results challenge the assumption that alert and warning signals should be “as salient as possible”^21,37^. Instead, we show that the design of alert signals might face a trade-off between capturing attention as quickly as possible and avoiding task interference. Importantly, this result is not limited to one specific parameter setting. We observe the trade-off for contrast as well as for duration of the visual alert, that is, for two entirely different stimulus dimensions. Increasing salience was beneficial up to a certain point, beyond which a further increase eventually became harmful. Adequate design of alert and warning signals therefore must identify the “sweet spot” at which attention is efficiently attracted *and* task interference is minimized. The observed interference is reminiscent of interference by irrelevant distractors^29–33^. There is, however, a critical difference: the alert signal provides valid spatial information and is therefore task-relevant—at least initially. Its role changes into that of a “distractor” only once attentional orienting is completed. The trade-off we observe bears some conceptual similarity to the trade-off between urgency and annoyance described for warning signals^3–6^, although the former trade-off operates on a rather sensory and the latter trade-off on a rather semantic level. The analogy to the urgency/annoyance trade-off can be pushed somewhat further if annoyance is understood as an umbrella term not only for emotional nuisances, but also for cognitive disturbances and interferences, as is typically the case in the context of auditory noise^46^.

Our approach to analyze the effect of salience separately on several dependent variables—time to fixation, alert reaction time, primary-task reaction time—allowed us to disentangle these different aspects of alert processing. As any modification to the design of an alert may be beneficial to some of these aspects and detrimental to others, understanding the effects on each provides a more comprehensive picture and eventually a more efficient guidance for alert-signal design. From basic research, there are several known effects that may contribute to the slowing down of the response at higher alert-frame salience, some of which are related to attention distraction, others to sensory interference:

(i) Attention might at first be directed to the alert frame rather than directly to the alert-task square, necessitating a second attentional shift. Shifting attention between objects incurs a cost, partly because attention spreads preferably within an object^47^, partly because disengagement from an object is costly^48^. If the cost of this second attentional shift depends on the frame’s salience,  this accounts for part of the observed detrimental effect of too high alert-frame salience. The analogy of the frame to a task-irrelevant distractor in attentional-capture tasks^35^ makes such a salience dependence of the cost likely. The difference in the fixation-to-response delay between most and least salient frame of 10 ms to 20 ms (Experiment 1) is consistent with the typical costs associated with the disengagement of attention from an object^48^ and with the increase in reaction observed when a distractor location contains a target feature (e.g. color)^49^. The interpretation as disengagement costs would also predict less interference if the alert frame is removed once it has served the function as cue (as observed in Experiment 2), and more interference if the frame’s salience is high specifically *after* the saccade (as observed in Experiment 4).
(ii) For the specific case of overt attention (i.e., attention related to gaze) in natural scenes, higher visual salience yields longer fixation durations^50–53^. Such prolonged fixations due to higher visual salience, which we indeed observed (Experiment 1), may contribute to the increased time from fixating the alert-task square to responding that counteract the speeded-up response.
(iii) Simultaneous contrast^54^ can make the interior of a brighter frame appear darker. The perceived contrast between the dark alert-task square and its background (which is the interior of the frame, Fig. 1c) would then be reduced as a consequence of higher alert-frame salience. Consequently, if the alert frame gets more salient, effects of simultaneous contrast would decrease the perceived contrast of the alert-task square relative to its background inside the alert frame. In a closely related interpretation, a salient alert square would act akin to a lateral mask, although effects of such masks are typically more prevalent in peripheral than in central vision^55^ and—given the size of the gap—processing of the alert square while it is still peripheral seems unlikely. Nonetheless, as alerts often have to be processed peripherally, such effects might even be stronger in practical applications. In either case, the task to detect the opening would become harder when the alert-frame gets brighter, and increased alert-frame salience would thereby increase the time from fixation to reaction.

We neither consider these explanations as an exhaustive list of possible mechanisms nor as mutually exclusive; rather, it is likely that a mixture of such attentional and sensory factors contributes to the observed effects. Importantly, the disadvantages of high salience in our paradigm are not caused by emotional or semantic factors that are already known to counteract beneficial effects of salience^1–6^.

It should be emphasized that—albeit abstract—our scenario resembles some critical aspects of alert-signal design. In particular, it distinguishes itself from most other attentional basic-research paradigms in terms of the role of the stimuli that might capture gaze and attention. In typical attentional paradigms, these roles are fixed throughout a trial: items are either task-relevant (e.g., as cues or targets) or task-irrelevant throughout. Specifically, in typical attentional-capture paradigms, the singleton distractor is an item known to be task-irrelevant, but still capturing attention. In our paradigm, the role of the alert signal changes during a trial: first it acts as a cue, i.e., is task-relevant and, for being 100% valid, always useful. Only once attention has been oriented towards the alert location, the alert signal becomes irrelevant and changes its role to being a distractor. That is, despite unchanged salience, the task relevance of the alert signal changes over the course of a trial. As such, we consider our paradigm as an extension of basic-research paradigms towards more realistic settings, where the same stimulus can adopt various roles with respect to the task even on a short time scale.

The huge body of research on attentional orienting and distraction in experimental psychology can be helpful to choose a set of feasible alert and warning signals, whose adequacy must then be tested in a task- and stimulus-specific manner. Although this research area has focused almost exclusively on task-irrelevant distractors, some general principles are likely transferable to contexts where the distractor is initially relevant (i.e., becomes a distractor only after it has subserved its function to direct attention to the alert). For instance, the amount of distraction is not dependent on physical stimulus properties per se, but on the discrepancy between the physical and the expected stimulus properties^56^. From classical experiments like the Eriksen flanker task^30^, it is evident that many factors contribute to the degree of distraction, such as the compatibility between the responses to target and distractor, the perceptual similarity between target and distractor, or the spatial layout of the display. Interference can involve high-level functions like language^33^, can act across modalities^57,58^ and may be decreased by perceptual load and increased by the need for cognitive control^59^. Systematic comparisons of auditory and visual distractors have revealed modality-specific differences in the ability to inhibit a distractor’s influence^60^ as well as in the time-course of distraction and of recovery from distraction^45^. These dependencies on overall context and on the users themselves are obviously critical in applied contexts. Future studies should clarify the extent to which the experimental results on irrelevant distractors transfer to situations in which the distractor itself bears relevant information at some point in time—that is, to the scenario we considered in this study. We argue that this step from task-irrelevant to task-relevant distractors is of high importance to real-world scenarios, where entities can dynamically assume relevance based on context.

Attentional capture can be reduced or absent, if the target-defining features are sufficiently distinct from those used in the singleton (e.g. the detection of color discontinuities is undisturbed by apparent motion^61^, although not entirely by abrupt onsets^62^). Hence, the relative similarity of the alert frame to the alert square may contribute to the distracting effect of the alert frame and therefore of visual salience in our paradigm. However, in practice, visual alerts and the required responses are often aligned (e.g., a relevant button that is illuminating), such that we consider our paradigm rather typical for such scenarios.

Although we used auditory and visual stimuli and found differences for the specific (visual) task, the present study was not designed to investigate general differences between modalities. To the contrary, we argue that optimal warning signals and therefore the optimal modality are specific to the task and thus to the application domain. There are some obvious differences between modalities that come into play. For example, if a task requires focusing gaze on a certain area and not all instrumentation can be brought into the line of sight, auditory warning signals are preferable over visual ones—with patient monitoring in intensive care providing a prototypical example^63^. Many studies that ask whether visual, auditory or tactile warnings are most efficient, focus on one particular scenario. The results typically depend critically on specific parameters^15,64, 65^, though there seems some consensus that combination of multisensory signals typically speeds up responses as compared to unimodal warnings^13,15, 66^. Interestingly, studies that compare the efficiency of warning signals across modalities usually base their conclusions on the immediate reaction to the warning, which is frequently measured in terms of reaction times or avoided errors. To reiterate, our results challenge such a single-measure approach. If—for example—we just looked at saccadic response times (time to fixation), we would arrive at entirely different conclusions compared to analyzing—say—only reaction times to the alert. Only by analyzing attentional effects in conjunction with their behavioral consequences—both to the alert and the primary task—we were able to obtain a full picture.

Irrespective of the mechanisms underlying the detrimental effects of high salience on the response, it remains likely that the differences between visual and auditory alert signals *in the present study* are at least partially attributable to the fact that both tasks (alert task and primary task) are visual. Although clearly beyond the scope of the present study, it remains an interesting and open question how visual salience may or may not affect auditory tasks^45^. As one hint that too high salience could be harmful for auditory tasks, Spence and Driver^67^ found a numerical decrease of performance during the discrimination of target sounds when a visual cue was added to a high-intensity auditory cue, while a low-intensity auditory cue remained unaffected. Moreover, the trade-offs between annoyance and urgency perception^3–6, 68^ and the sweet spot for the combination of semantic urgency and sound level for an optimal warning^25–27^ are particularly evident in the auditory case. Importantly, we do *not* argue that auditory alerts are *generally* preferable over visual alerts. Rather, benefits and costs of salience will most likely depend on the tasks to be executed. This highlights the need for a *task-specific* or *application-specific* design of alert and warning signals and underlines the benefits of carefully dissociating their separate effects on attentional orienting, on the execution of the alert task, and on conducting the primary task.

Our results are not informative with respect to cross-modal effects because we used congruent audio-visual signals throughout. Moreover, the alert frame coincided with the alert stimulus location, while the alert tone—presented through headphones –was congruent only with respect to the side, but not with respect to the exact location. This is uncritical to the usage of bimodal alerts in the present paradigm, as even spatially uninformative cues can yield cross-modal benefits for warning signals^69^, notwithstanding additional benefits of cross-modal spatial congruency^70,71^. Besides spatial congruency, the relative timing of cue(s) and target contributes to successful cross-modal warning^67^. Timing is also crucial within a modality: initially beneficial effects of exogenous cues revert after some hundred milliseconds^72^, an effect known as inhibition of return^73^. It should be noted, however, that unlike in mere cueing tasks, in our task the first reaction to the alert must be an eye movement: due to the small size of the gap, processing of the “target” (the alert square) is only possible once the eyes have landed on the alert-task square. In this respect, our task differs from tasks that only allow covert attentional shifts (i.e., without eye movement) and from settings in which processing can start prior to shifting the gaze. Nonetheless, the issue of the optimal spatial and temporal relation among visual cue (alert frame), auditory cue (alert tone) and target (alert-task square) remains an interesting issue for future research. This is especially so in applied contexts, where an early warning that increases alertness, but not necessarily guides attention, in some circumstances is more effective than a late warning that guides selective attention to a potential hazard^24^. This argues for further dissociation of the non-selective “alerting” component of a signal and the attentional “guidance” aspect of a warning signal in future research. The practical relevance of our specific design might be challenged on the grounds that contemporary alert or warning signals rarely coincide spatially with the actual hazard, emphasizing the alerting over the guidance aspect. However, with ever increasing possibilities in augmented reality, e.g., head-up displays in cars, this balance is currently shifting. As one example, the study by Werneke and Vollrath^24^ also included a condition in which the warning signal was superimposed on the hazard, which in their case was less effective than an earlier warning. Such results and the increasing technological possibilities to “blend” warnings into actual reality, will also increase the direct applicability of our laboratory design. Along similar lines, it could be argued that—unlike in many applied scenarios – our participants do not react to avoid a negative outcome, but rather respond as quickly as possible, which again has more resemblance of a classical cueing task than to alerts in applied scenarios. However, in many applied scenarios the goal is also a quick response (e.g. braking for collision avoidance in driving).

Some choices regarding the experimental design were common across all four experiments: the same central task had to be performed, all alerts were bi-modal, and alert prevalence was at 12.5%. Unlike typical dual-task designs, we enforced absolute priority for the alert task, which had to be completed before continuing with the primary task. Nonetheless, we could quantify “missed” alerts by the number of alert-task intrusions and found this number to be low across all experiments (cf. Table 1). It is likely that the alert’s bi-modality and the comparably high alert prevalence contributed to this low miss rate. Bimodal signals are known to be more effective in alerting a user engaged in a primary task than unimodal signals, especially if the primary task induces high perceptual load^74–76^. This is also in line with our observation that intrusions are most frequent when visual and auditory salience are low (cf. part 3 of the Supplementary Material). By design, the onset of the alert task was always simultaneous with the primary task (and therefore with the end of the response—the button release—to the previous primary task); it might be an interesting issue for future research to vary the timing between the two and to provide feedback on intrusions at various points in the trial to effectively reduce the distraction by the primary task (cf. Ref.^77,78^). Since our aim was to isolate possible negative effects of the alert signal’s salience even under favorable conditions, we did not manipulate load, but kept the primary task simple and constant. Given the detrimental effects of low prevalence on attention tasks, such as visual search^79^, the rather high alert-trial prevalence probably also contributed to the low number of missed alerts. In line with low-prevalence effects, the time course over a block (part 4 of the Supplementary Material) shows that the first alert in a block evokes substantially higher reaction times than later alert trials, suggesting that the first alert frame may capture attention similar to features that occur surprisingly^80,81^. Moreover, attentional capture by abrupt onsets is reduced or abolished when these onsets are frequent, while the same onsets can capture attention when presented infrequently^82^. In this context, it is an interesting issue for further research, whether repeated exposure to high-salience alerts reduces their distracting effects in a similar manner as suppressing saccades towards repeated task-irrelevant distractors is learnt^83–85^. For the envisioned applications, however, our chosen prevalence is rather high compared to real-world scenarios^86^, such that alerts are likely to operate in a lower-prevalence regime. It is conceivable that higher task difficulty, e.g. as a consequence of lower alert-trial prevalence or a harder central task, would shift the sweet spot of optimal salience to higher levels. The central argument—maximizing salience, i.e., optimizing the *orienting* to the alert, does not imply a maximally efficient *response* to the alert—remains, however, unchallenged by such quantitative shifts. In this context, it is also important to note that we did not focus on the question whether alerts were missed or noticed, but on the speed at which a reaction occurred. The assertion that for practical applications alert signals might need adaptation to alert prevalence, the current load, or to the current state of the user in general, highlights the need for task-specific and application-specific design considerations even further.

Besides their current state, characteristics and attitudes of the expected “receiver” (i.e., the user to be warned or alerted^87^) play an important role in alert and warning signal design. This includes sensory aspects, such as visibility and noticeability, especially when age-related characteristics are concerned^1^. Our study was not designed to assess such characteristics, as we recruited a rather homogeneous sample (young healthy adults, mostly university students). Nonetheless, we performed an explorative analysis of the relation between our dependent variables and age, which did not reveal any consistent patterns in this sample regarding timing, though there were some isolated negative correlations of reaction time with age. Interestingly, in our specific group of participants, the ability to discriminate alert trials from non-alert trials showed some dependence on age, with a small positive effect of age on this ability. The details of this analysis are given in part 5 of the Supplementary Material, but it should be stressed that our experiments were *not* designed to test individual differences and that thus age might well be confounded with other factors (e.g., the level of experience with psychophysical experiments). Relating the location of the sweet spot for alert-signal design to individual characteristics, in particular to age, might be an interesting avenue for further research.

Detrimental effects arising from alert signals that are too salient have been described in several contexts. These include the inappropriateness of extreme loudness or brightness that impair sight or hearing or yield a startle response^2^, interference with other signals that fail to gain appropriate priority^2^, the aforementioned annoyance-urgency trade-off that is parameterized by salience^27,68^, increase of perceived workload with louder signals^88^, and the deactivation of alerts that are perceived as disturbing^2^. The present paper deals with a related but distinct issue: negative effects of too high salience arise for an alert that has *no concurrent task*, requires only a *single transient response,* always has *priority* (i.e., its urgency is constant), and is not hazardous or disabling in itself. The detrimental effect of salience in our case arises from a trade-off between attentional orienting towards a cue, which is quantified by gaze tracking, and subsequent interference of the very same cue with the task. We believe that the results are of relevance to a variety of applications and provide a further challenge to the notion that a highly salient signal is always best suited to induce a maximally rapid and adequate response.

## Methods

### Participants

Seventy-eight healthy adult volunteers participated in the experiments and were included in the analysis, 20 in Experiment 1 (13 female, 7 male, age 21–35 years, mean: 25.0 years, SD: 3.6 years), 19 in Experiment 2 (9 female, 10 male, age 19–30 years, mean: 23.3 years, SD: 3.3 years), 20 in Experiment 3 (10 female, 10 male, age 18–39 years, mean: 24.6 years, SD: 5.6 years), and 20 in Experiment 4 (11 female, 9 male, age 20–31 years, mean: 24.1 years, SD: 3.42 years). One participant of Experiment 1 also participated in Experiment 3, otherwise participant groups were disjoint. Six further participants were excluded as they could not complete the experiment because of technical failure of the eye-tracking system. One further participant of Experiment 2 was excluded from analysis after experiment completion as the primary task performance showed less than 70% correct responses. Due to technical failure, for one participant of Experiment 2 the last 62 trials of one block (of which 7 were alert trials) were not recorded; the remaining 5058 trials (633 alert trials) were unaffected.

All participants gave written informed consent prior to participation. All procedures conformed to the Declaration of Helsinki; the Institutional Review Board (*Chemnitz University of Technology, Ethikkommission Fakultät für Human- und Sozialwissenschaften*) decided that no in-depth evaluation of the ethical aspects was required (case no.: V-148-AB-Alarm-01072016). The IRB application included the choice of 20 participants per experiment, which had been estimated to provide sufficient power based on previous studies on saccadic latencies.

### Setup

Experiments were conducted in a sound- and light-attenuated room. Visual stimuli were presented on a VIEWPixx/3D Full monitor (VPixx Technologies Inc., Saint-Bruno, QC, Canada). Auditory stimuli were presented using the monitor’s in-built sound card through calibrated Sennheiser HD 25-1 (70 Ω) headphones. Resolution of the screen was 1920 × 1080 pixels, the frame rate 120 Hz. Behavioral responses were recorded with a ResponsePixx (VPixx Technologies Inc.) 5-button box. The arrangement of buttons clearly identified up, down, left and right button (Fig. 1a), the middle button was not used. Left/right responses were given with the left and right index finger, respectively, up/down responses with either index finger as chosen by the participant. Eye movements were recorded for the left eye throughout the experiments with an Eyelink-1000 Plus (SR Research Ltd, Ottawa, ON, Canada) eye-tracking device at 1000 Hz. Prior to each block, the eye-tracker was calibrated and validated with a 5-point calibration. Saccade and blink detection used the manufacturer’s algorithm with saccade thresholds at a velocity of 35°/s and an acceleration of 9500°/s^2^. Participants were seated in 57 cm distance from the screen. Their head was stabilized with a padded chin rest and forehead rest that are part of the eye-tracking device. Stimuli were generated and presented using Matlab 8.5.0 (Mathworks, Natick, MA) with its Psychophysics^89,90^ and Eyelink^91^ toolbox extensions.

### Stimuli

Visual stimuli were presented on a grey background that spanned the whole screen (10 cd/m^2^; 49 × 29 degrees of visual angle). Task-relevant squares were black (< 0.1 cd/m^2^), and 0.33 degrees of visual angle (12 pixels) wide. Primary-task squares were presented centrally, alert-task squares were presented 10 degrees (360 pixels) laterally to either side. Luminance levels of the alert frame were 11.0, 11.9, 13.6, 16.7, 22.7, 33.9, 55.1 or 95.0 cd/m^2^, which correspond to logarithmically-spaced Weber contrasts between 0.1 and 8.5. The alert frame was 1.5 degrees (54 pixels) on the inside and 2.1 degrees (76 pixels) on the outside. Alert sounds were sinewave tones of 512 Hz with 5-ms raised-cosine onset and offset amplitude ramps and were presented monaurally at the side of the square. The tone had an A-weighted sound pressure level of 54, 59, 64, 69, 74, 79, 84, or 89 dB(A).

### Gaze-contingent stimuli in Experiment 4

Experiment 4 used a gaze-contingent paradigm, in which the luminance of the alert frame changed once an eye movement was initiated towards the alert-task frame. This change was initiated when the eye position was more than 2 degrees of visual angle horizontally off-center towards the alert-task square side. Once this threshold was reached, the change of the display took place within less than 11 ms (maximally 2 ms delay of the eye-tracking signal plus maximally 8.3 ms delay between two frames at the 120 Hz frame rate). We verified offline that the display change had been completed well before the eye landed.

### Distribution of alert trials

The 64 alert trials of each block were placed at random with equal probability at each trial with the following restrictions: (i) the first and the last trial could not be an alert trial, (ii) between two alert trials, there was at least one no-alert trial. Half of the alerts in each condition and block were presented on the right side, half on the left. In each block of each experiment, each condition occurred equally often. The assignment of condition to alert trial within a block was random.

The initial block without alert trials was not included in any analysis except for part 4 of the Supplementary Material, where initial learning of the task is analyzed.

### Instruction

Participants were instructed to perform the tasks as quickly as possible without sacrificing accuracy. They were informed that if an alert occurred, the alert task had to be handled before they could proceed with the primary task.

### Statistical analysis

All data pre-processing was done in Matlab. Statistical analysis was performed using the R software package version 3.4.4^92^. Data and analysis files are available at 10.5281/zenodo.10938569. In Experiment 1, we tested for effects of either contrast or sound level on each of the dependent variables by using 1-factor repeated-measures ANOVAs. Moreover, we performed a linear regression to the individual data for each dependent variable as function of visual salience (contrast) or auditory salience (sound level). The slope, measured in milliseconds per salience step (ms/step), of this function captures the increase or decrease of the dependent variable with salience. For each dependent variable, slopes were compared to 0 with a two-sided one-sample t test, as well as between the visual and the auditory conditions by means of a two-sided paired t-test. Since all visual conditions were accompanied by the lowest sound-level alert tone and in turn all auditory conditions were accompanied by the lowest-contrast alert frame, the lowest contrast and lowest sound-level condition were identical for auditory and visual conditions. As auditory and visual conditions were analyzed separately, the total amount of data from this condition (i.e., twice the data of the other conditions) was used in the analyses of both modalities.

For Experiment 2, we tested the dependence of each dependent variable on contrast and duration by a 2-factor repeated-measures ANOVA with the factors contrast (2 levels) and duration (8 levels). For Experiment 3, we analogously tested the dependence of each dependent variable on sound level (2 levels) and sound duration (8 levels). Follow-up paired t-tests were applied when appropriate based on the ANOVA results and are reported in part 2 of the Supplementary Material. For Experiment 4, we tested the dependence of each dependent variable on sound level, contrast level before the saccade, and contrast level after the saccade by a 3-factor repeated measures ANOVA, with post-hoc tests as appropriate. For the ANOVAs in all experiments, Greenhouse–Geisser (GG) corrections for non-sphericity were applied for factors with more than two levels. Adjusted p-values, uncorrected degrees of freedom and GG-epsilon (ε) are reported. As measure of effect size that captures the within-subject design, generalized eta-squared (η_G_^2^) is provided; following Bakeman^93^, effect sizes can be classified as small for η_G_^2^ = 0.02, as medium for η_G_^2^ = 0.13 and as large for η_G_^2^ = 0.26. In addition, partial η_p_^2^ is reported for two-factor and three-factor ANOVAs, as well as η^2^ for one-factor ANOVAs (which for this case is equivalent to η_p_^2^). All statistical tests were two-tailed; to improve readability we nonetheless describe the results as directed where appropriate.

### Supplementary Information

Supplementary Information.

## Data Availability

Data and analysis files are available in the zenodo repository 10.5281/zenodo.10938569.﻿
