# Supplementary material for: High visual salience of alert signals can lead to a counterintuitive increase of reaction times
Source: Sci Rep. 2024 Apr 17;14:8858. doi: 10.1038/s41598-024-58953-4 (PMC11024089; doi:10.1038/s41598-024-58953-4)
Supplement: Supplementary file 1 — Supplementary Information. [file 41598_2024_58953_MOESM1_ESM.pdf]

Supplementary Material, Part 1: Salience models

To verify that our experimental manipulation of visual contrast indeed affects saliency as intended, we use 3 common models of low-level salience to compute salience across the stimuli for the 8 different contrast levels. Specifically, we computed the saliency map[S1], graph-based visual salience (GBVS[S2]) and the spectral salience[S3]. For the former two models we used the implementation of Harel and colleagues[S2], for the latter the implementation of Schauerte and Stiefelhagen[S4]. As the stimuli are symmetric relative to the image center and none of the models has any off-center spatial bias, we restricted this analysis to alert squares appearing on the left-hand side open to the top and primary task squares open to the right. Models were used in their default settings and maps were scaled back to the original image size for analysis. For the spectral model, the map was computed on a 10th of the image size, in accordance with the typical settings of the other models. In line with the typical procedure when using salience maps, the actual stimuli were put into the models (i.e., the pixel values used for presentation), such that the values were not linear in luminance, but scaled with the screen's gamma. This may affect the precise functional form of the salience values' dependence on "contrast", but a monotonic relation will stay monotonic. As by the Harel et al. implementation, saliency maps and GBVS maps were scaled to a dynamic range from 0 to 1, while spectral salience was scaled to the maximum across all maps for display. We compared the salience of the alert-task square S_A to the primary-task square S_P by computing the mean across the 16x16 pixels of the map corresponding to the respective square. Using these values, we computed the global salience index (GSI[S5]), which has been suggested earlier[S6] to compare targets with distractors, as $(S_A-S_P)/(S_A+S_P)$. This value is bound between -1 and 1, where 1 means maximal salience at the alert task square, -1 maximal salience at the primary task square. For all models, the alert-task square and the primary-task square are more salient than the background (Fig. S1). Importantly, with increasing contrast the alert-task square becomes relatively more salient compared to the primary-task square, and the GSI increases monotonically with contrast (except for the lowest levels of the spectral salience measure). This shows that the intuitive definition of increasing salience by increasing contrast is compatible with standard models of low-level salience.

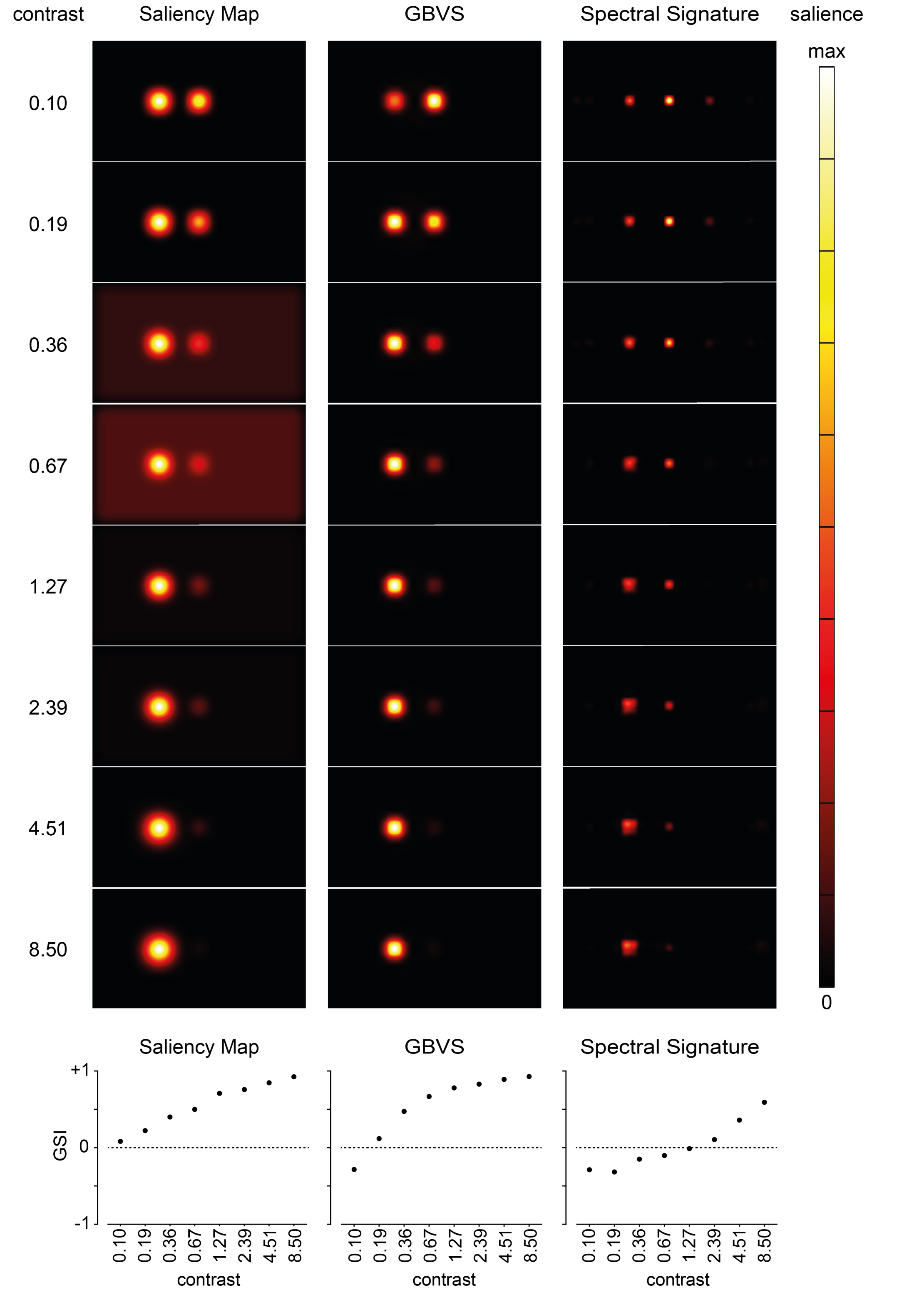

Supplementary Figure S1. Salience models. Maps resulting from the saliency map model, GBVS and spectral signature for the eight stimuli with alert square and frame on the left-hand side of the stimulus. Contrast increasing from top to bottom. The same scale is applied to all maps of each model. GSI as function of contrast given in bottom row.

Supplementary Material, Part 2: Tables for Follow-up Analyses

For variables for which a main effect of contrast or sound pressure level was observed at a 5% significance level in Experiment 1, pairwise follow-up tests were conducted, which are reported in Table S1.

Follow-up tests for the analyses of Experiment 2 are reported in Table S2. If there was a main effect of presentation duration, but no interaction with salience, the data were averaged across salience levels and pairwise follow-up tests were conducted.

Supplementary Table S1: Experiment 1, p-values of all pair-wise follow-up tests for variables for which a significant main effect of contrast or sound pressure level was observed. Red p-values indicate significance at a 5% level after Bonferroni-Holm correction for the 28 tests conducted in the respective table; blue values indicate uncorrected significance.

Table S1A: time to fixation, visual

contrast	0.10	0.19	0.36	0.67	1.27	2.39	4.51	8.50
**0.10**		.030	.004	<.001	<.001	<.001	<.001	<.001
**0.19**			.298	.060	.050	.002	.022	.003
**0.36**				.292	.152	.003	.023	.002
**0.67**					.926	.107	.360	.086
**1.27**						.030	.125	.012
**2.39**							.457	.480
**4.51**								.184
**8.50**								

Table S1B: time to fixation, auditory

dB(A)	54	59	64	69	74	79	84	89
**54**		.080	<.001	.001	.002	<.001	<.001	<.001
**59**			.372	.030	.087	.009	.077	<.001
**64**				.215	.126	.114	.172	<.001
**69**					.928	.620	.919	.017
**74**						.698	.835	.025
**79**							.829	.038
**84**								.042
**89**								

Table S1C: RT alert, auditory

dB(A)	54	59	64	69	74	79	84	89
**54**		.054	.004	.002	.007	.084	.002	<.001
**59**			.144	.030	.188	.593	.042	<.001
**64**				.509	.997	.478	.260	.048
**69**					.376	.226	.761	.039
**74**						.491	.307	.049
**79**							.082	.017
**84**								.233
**89**								

Supplementary Table S2: Experiment 2, p-values of all pair-wise follow-up tests for variables for which a significant main effect of duration was observed. Red p-values indicate significance at a 5% level after Bonferroni-Holm correction for the 28 tests conducted in the respective table; blue values indicate uncorrected significance.

Table S2A: time to fixation

duration	25 ms	50 ms	100 ms	200 ms	300 ms	400 ms	600 ms	800 ms
25 ms		.005	.111	<.001	<.001	<.001	.005	<.001
50 ms			.913	<.001	<.001	<.001	<.001	<.001
100 ms				<.001	<.001	<.001	<.001	<.001
200 ms					.585	.242	.019	.560
300 ms						.695	.187	.843
400 ms							.148	.477
600 ms								.016
800 ms								

Table S2B: RT alert

duration	25 ms	50 ms	100 ms	200 ms	300 ms	400 ms	600 ms	800 ms
25 ms		.997	.350	.156	.276	.321	.049	.405
50 ms			.249	.150	.379	.349	.038	.415
100 ms				.027	.048	.817	.250	.964
200 ms					.487	.004	.007	.063
300 ms						.046	.003	.038
400 ms							.538	.807
600 ms								.255
800 ms								

Table S2C: Fixation-to-response delay

duration	25 ms	50 ms	100 ms	200 ms	300 ms	400 ms	600 ms	800 ms
25 ms		.466	.913	.618	.049	.006	.002	.019
50 ms			.286	.217	.021	<.001	.001	.002
100 ms				.771	.026	.002	<.001	.027
200 ms					.130	.002	.019	.046
300 ms						.122	.026	.321
400 ms							.759	.907
600 ms								.682
800 ms								

Table S2D: Fixation duration

duration	25 ms	50 ms	100 ms	200 ms	300 ms	400 ms	600 ms	800 ms
25 ms		.808	.376	.018	.002	.032	.003	.008
50 ms			.212	.024	.002	.022	.004	.009
100 ms				.001	.000	.002	.001	.002
200 ms					.041	.773	.510	.367
300 ms						.442	.434	.724
400 ms							.817	.599
600 ms								.782
800 ms								

Supplementary Material, Part 3: Behaviour in alert trials

For alert trials, we analysed how the probability for alert-task intrusions (i.e., mistakenly responding to the primary task first despite an alert) depended on the independent variables in each of the four experiments. For Experiment 1, we found a dependence both on visual ($F(7,133) = 4.40$, $p = .002$, $\varepsilon = 0.60$, $\eta_G^2 = .115$, $\eta^2 = .188$) and on auditory salience ($F(7,133) = 5.80$, $p < .001$, $\varepsilon = 0.69$, $\eta_G^2 = .159$, $\eta^2 = .234$) with a near monotonic dependence on salience in both modalities (Fig. S2a,b). That is, with increasing salience – contrast or sound pressure level – the probability to miss an alert decreased.

In addition, we applied the same exclusion criteria as in the analyses of the main text except alert-task correctness (i.e., excluding alert trials with alert-task intrusions, fixation errors, primary-task intrusions or primary-task errors) and computed the percentage of errors resulting from responding incorrectly to the alert (i.e., pressing the down button instead of the up button or vice versa) in these otherwise correct trials. The fraction of these errors, which we refer to as alert-response errors throughout this part of the supplement, was near floor and did not systematically depend on salience (Fig. S2c, d), neither for contrast ($F(7,133) = 0.684$, $p = .609$, $\varepsilon = 0.59$, $\eta_G^2 = .027$, $\eta^2 = .035$; Fig. S2c) nor for sound pressure level ($F(7,133) = 1.56$, $p = .195$, $\varepsilon = 0.57$, $\eta_G^2 = .047$, $\eta^2 = .076$; Fig. S2d).

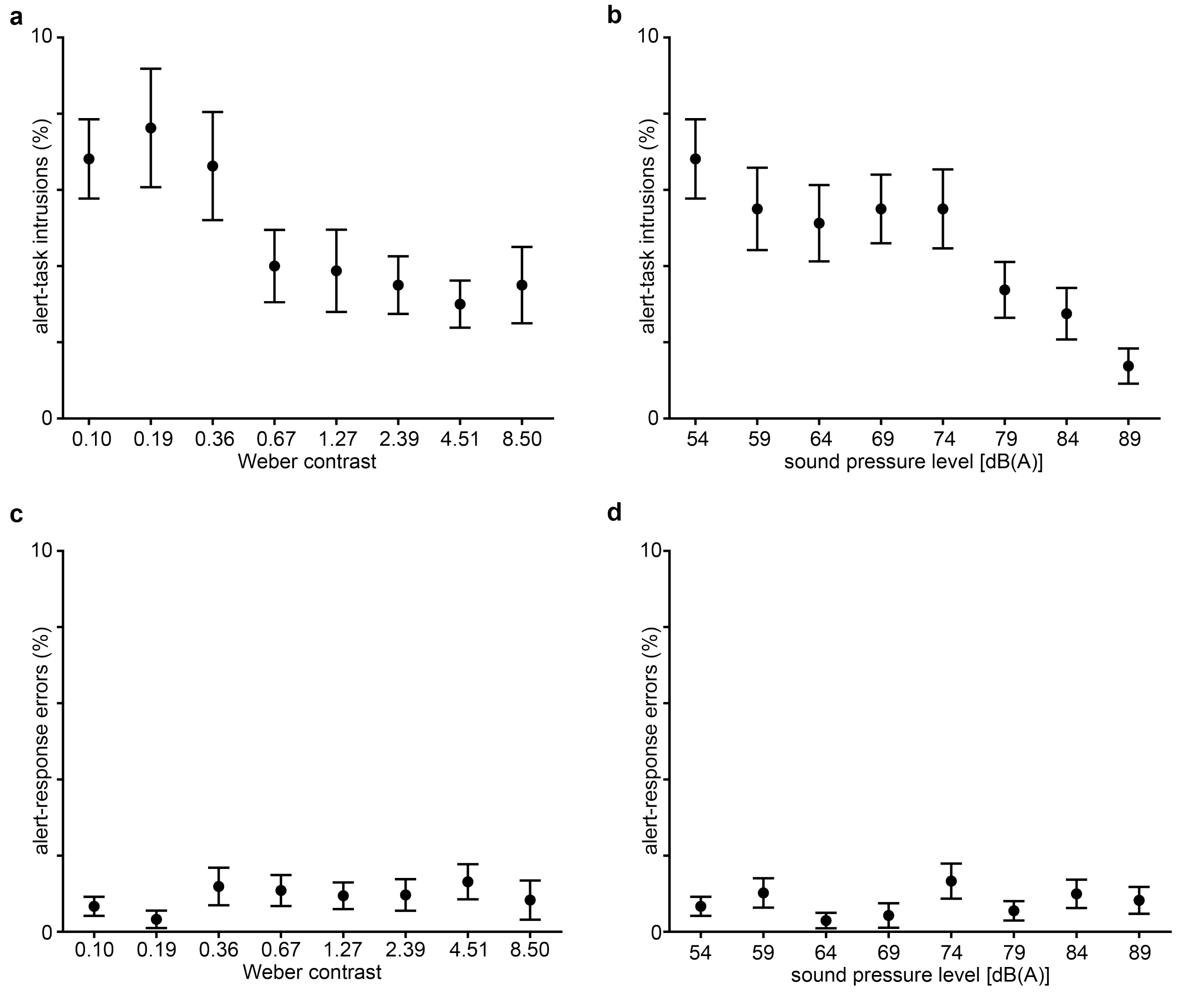

Supplementary Figure S2. Dependence of alert-trial performance on salience. a-b) Percentage of alert trials with alert-task intrusions in Experiment 1 as a function of contrast (a) or sound pressure level (b). **c-d**) Percentage of alert trials in which the response to the alert was incorrect, given no other errors occurred in this trial ("alert-response error"), as a function of contrast (c) or sound pressure level (d).

In Experiment 2, fewer alert-task intrusions were observed at the higher salience level than at the lower salience level ($F(1,18) = 18.7$, $p < .001$, $\eta_G^2 = .028$, $\eta_p^2 = .510$). There was no main effect of alert-frame duration on alert-task intrusions ($F(7,126) = 1.48$, $p = .209$, $\varepsilon = 0.65$, $\eta_G^2 = .009$, $\eta_p^2 = .076$), nor an interaction between the factors ($F(7,126) = 0.435$, $p = .802$, $\varepsilon = 0.63$, $\eta_G^2 = .002$, $\eta_p^2 = .024$). We did not observe any main effects of salience, or alert-frame duration on alert-response errors nor an interaction between the factors (all $p > .193$).

In Experiment 3, fewer alert-task intrusions were observed at the higher salience level than at the lower salience level ($F(1,19) = 12.7$, $p = .002$, $\eta_G^2 = .028$, $\eta_p^2 = .401$). There was no main effect of alert-tone duration on alert-task intrusions ($F(7,133) = 0.69$, $p = .611$, $\varepsilon = 0.62$, $\eta_G^2 = .004$, $\eta_p^2 = .035$), nor an interaction between the factors ($F(7,133) = 2.10$, $p = .082$, $\varepsilon = 0.63$, $\eta_G^2 = .010$, $\eta_p^2 = .099$). For alert-response errors we found a trend towards a main effect of salience level ($F(1,19) = 3.90$, $p = .063$, $\eta_G^2 = .011$ $\eta_p^2 = .170$), but no main effect of duration ($F(7,133) = 0.28$, $p = .925$, $\varepsilon = 0.74$, $\eta_G^2 = .005$, $\eta_p^2 = .015$). There also was an interaction between the factors ($F(7,133) = 2.69$, $p = .033$, $\varepsilon = 0.62$, $\eta_G^2 = .05$, $\eta_p^2 = .124$). Inspection of the results pattern suggests that *fewer* errors occurred for the quieter sound, in particular if it was brief in duration. It is possible that this is an effect of excluding alert-task intrusions from this analysis, which may bias alert responses towards more correctness if an alert that is difficult to detect is not missed. Nonetheless, the pattern of results, in particular with respect to intrusions (i.e., initially missed alerts), in Experiments 2 and 3 is consistent with Experiment 1 regarding salience, and no effects of alert duration on intrusions or alert-response correctness were found.

In Experiment 4, higher auditory salience led to fewer alert-task intrusions than lower auditory salience ($F(1,19) = 11.3$, $p = .003$, $\eta_G^2 = .015$, $\eta_p^2 = .373$). Similarly, higher visual salience before the saccade led to fewer alert-task intrusions than lower visual salience before the saccade ($F(1,19) = 12.7$, $p = .002$, $\eta_G^2 = .009$, $\eta_p^2 = .400$). There also was an interaction between these two factors ($F(1,19) = 6.87$, $p = .017$, $\eta_G^2 = .003$, $\eta_p^2 = .266$) with the highest number of alert-task intrusions when auditory salience and visual salience before the saccade both were low. There was no effect of visual salience after saccade nor any further interactions (all $p > .268$). No main effects or interactions were observed for alert-response errors (all $p > .174$).

Consistent across experiments, we observe more alert-task intrusions for lower salience (visual or auditory) – that is, more alerts are missed if salience is low. However, once the alert is successfully detected[1], there is little to no effect of salience on the correctness of the response to the alert.

[1] Note that for consistency with the analyses with the main text, we also excluded alert-trials with primary-task intrusions and primary-task errors when calculating alert-response errors. Omitting this exclusion criterion (i.e., excluding only trials with alert-task intrusions or fixation errors for computing the alert-response errors), does not affect the pattern of results.

Supplementary Material, Part 4: Time course

Although our experiments were not designed to analyse learning or temporal evolution of our effects, it is relevant to check whether there are general effects on performance and reaction times over time (e.g., whether the effects “wear off”). We deliberately aggregate over experiments and conditions for this analysis rather than segregating by salience level or any other independent variable, as these were not counterbalanced for order.

Primary task

Since this analysis is concerned only with no-alert trials, we aggregated over all four experiments. In all paradigms the first block was used as training without alert trials. Participants learnt the task quickly, reaching an about constant performance level after about 20 trials (Fig. S3a). We fitted each individual’s reaction time data with an exponential of the form $A \exp(-(t-1)/\tau) + b$, where the parameter b is the steady state reaction time reached after training, τ the time constant (in number of trials), t the trial number and A+b the reaction time prior to any training. With the exception of one participant in Experiment 3, whose reaction time decreases throughout the training block, all other participants are fit well by this function. For those 78 participants, the maximum value of τ is 20.9 trials, while 52 are close to their asymptotic performance after the first trial (i.e., have $\tau < 1$; Fig. S3b). This means that τ is always very small compared to the number of training trials (512). Hence, we can safely assume that all except one of the participants have reached a steady-state performance far before the end of the training block, even though there might be a slight increase in reaction time towards the end of the block not captured by the fit. The steady-state reaction time (fit parameter b) varies considerably across participants with a mean of 416 ms and a standard deviation of 76 ms (Fig. S3c). The single participant not fitted well with the exponential decay, over the last 100 training blocks has an average reaction time of 600 ms, which is well within this range.

As a second step, we analysed whether the primary-task reaction times for correct trials (excluding alert trials) changed over the course of blocks. We found a main effect of block number on the median reaction time ($F(9,702) = 7.13$, $p < .001$), which numerically decreased till block 6 until it levelled off (Fig. S3d). Similarly, the factor block had a significant effect on the fraction of correct trials ($F(9,702) = 8.56$, $p < .001$) with performance getting *worse* from block 1 through 9 (Fig. S3d). While the magnitude of these effects is minuscule (the average percentage of correct trials ranging from 95.9% in block 1 to 93.8% in block 9, and the median RT ranging from 369 ms in the first block to 354 ms in the sixth block), participants - on average - seem to trade some accuracy for speed as the experiment progresses. While this is an interesting - though not unexpected - observation, it has little bearing on the main results, as each condition occurs equally frequently in each block. Over the course of a block, accuracy in no-alert trials on average decreased (Fig. S3e). When excluding the first trial from analysis, which on average took excessively long (mean over the median reaction time per participant: 708ms), we found that over the course of a block reaction time in no-alert trials increased (Fig. S3f). This also holds for individuals: the accuracy for a given trial number (averaged across blocks) had a negative rank (Spearman) correlation with trial number for 72/79 participants (Fig. S3g) and the reaction time (again excluding the first trial and taking the median across blocks) had a positive linear (Pearson) correlation with trial number in 75/79 participants (Fig. S3h). In sum, while over the course of the whole experiment, participants get faster at the cost of reduced accuracy, within a block, the bulk of participants gets worse and slower, but all these effects are of modest size.

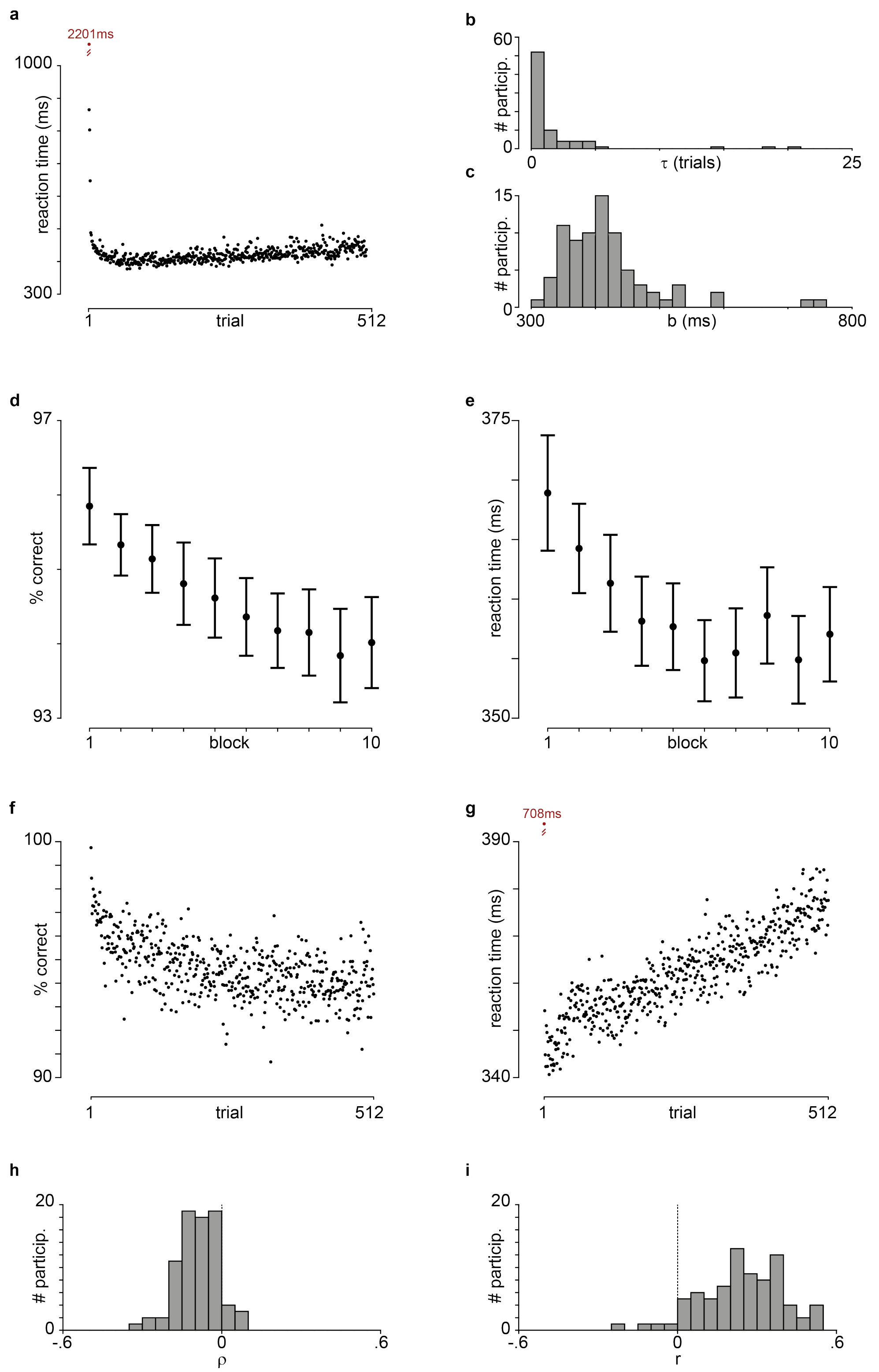

Supplementary Figure S3. Performance in primary task over time. a) Reaction time in training block by trial number, mean across participants, **b)** histogram of individual fits for time constant of learning, **c)** histogram of individual fits for asymptotic reaction time, **d)** percentage of correct no-alert trials as a function of block number (mean and s.e.m. across participants) **e**) primary-task reaction time in correct no-alert trials as function of block number (median across trials per participant, mean and s.e.m. across participants), **f**) percentage of correct no-alert trials as a function of trial number (mean across participants, errorbars omitted for display clarity), **g**) reaction time in correct no-alert trials as function of trial number (median across blocks per participant, mean across participants, errorbar omitted for display clarity), **h**) histogram of correlation coefficients for the Spearman correlation between trial number (1..512) and the percentage of correct primary task responses in the respective trial across blocks in each individual; **i)** histogram of correlation coefficients for the Pearson correlation between the trial number (excluding the first trial, 2..512) and the median primary-task reaction time across blocks for the respective trial in correct no-alert trials in each individual.

Alert task

To estimate how the performance in alert trials develops within a block and over the course of the experiment, we aggregated all alert trials across all experiments irrespective of condition. We consider how the probability to execute an alert trial completely correctly (i.e., no intrusions, no fixation error, responses to alert task and primary task correct; 100% minus “any error” of Table 1) varies over blocks and over trials within a block. The fraction of correct alert trials showed a dependence on block number ($F(9,702) = 16.10$, $p < .001$) with an overall performance increase from early to late blocks (Fig. S4a). Alert reaction time, in turn, depended on block number ($F(9,702) = 3.83$, $p < .001$) with a near monotonic speed-up over the course of the experiment (Fig. S4b). Hence, while the speed-up follows the same trend as the primary tasks, there is no speed-accuracy trade-off over the course of the experiment. There is a decline in correctly executed alert trials over the course of a block, although the first alert trial in a block is considerably more error-prone than all the remaining alert trials (Fig. S4c). For the alert reaction time we observe a similar pattern as for the primary task: the first 2 to 3 alert trials in a block are substantially slower than the following ones, while there is a slight slowing over the course of the block (Fig. S4d). Note that – as in the main analyses – only completely correct alert trials are included in the analysis. The qualitatively observed patters are confirmed by computing correlations between trial number and the performance measure in each individual (excluding the first alert trial in each case). Despite considerable variability across individuals (Fig. S4e), the mean Spearman correlation (-.080) is shifted slightly to the negative on average with the mean across individuals significantly smaller than 0 ($t(78) = 4.13$, $p < .001$). Similarly, reaction times tended to increase over the course of a block with a mean Pearson correlation of .09 across individuals (Fig. S4f; $t(78) = 3.69$, $p < .001$).

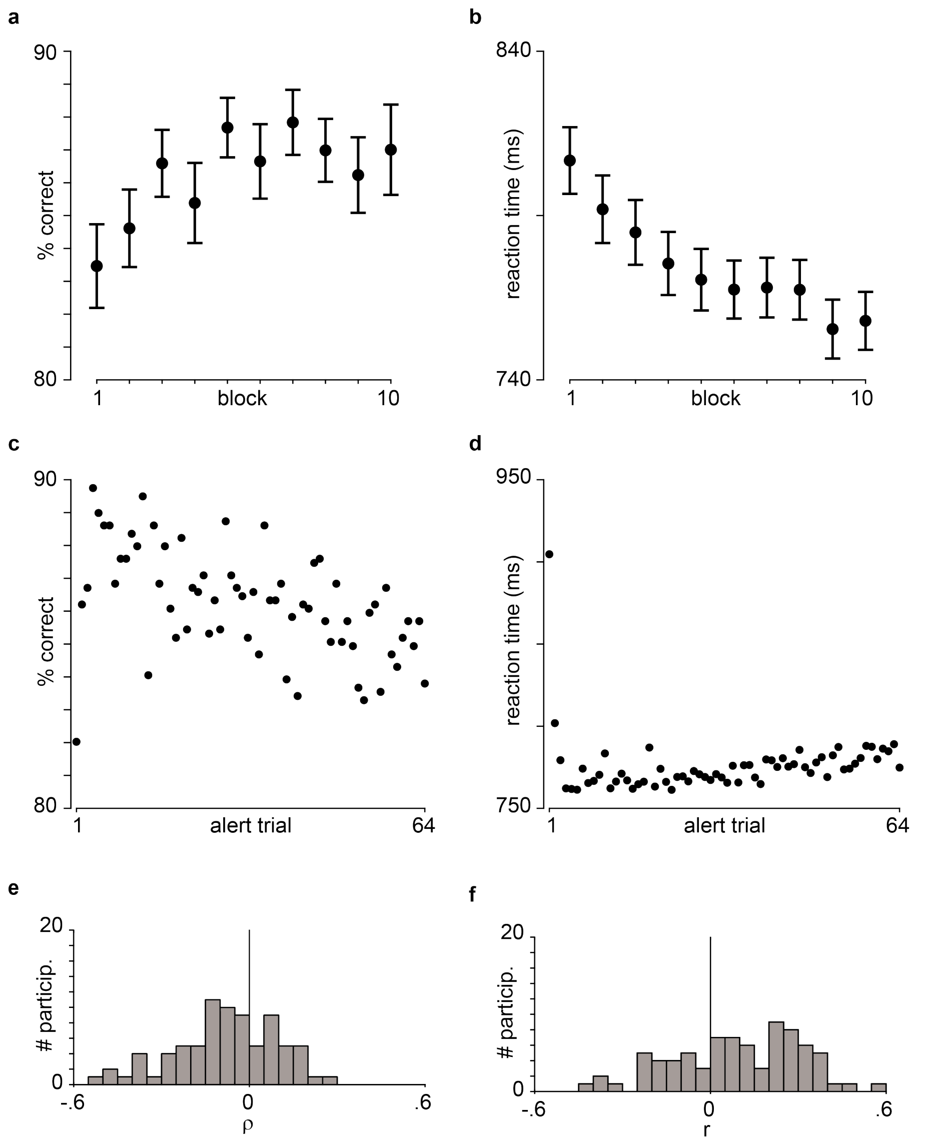

Supplementary Figure S4. Performance in alert task over time. a) Percentage of completely correct alert-trial responses as a function of block number (mean and s.e.m. across participants), **b**) reaction time as function of block number (median across trials per participant, mean and s.e.m. across participants), **c**) Percentage of completely correct alert trials as a function of alert-trial number (mean across participants; errorbars omitted for display clarity), **d**) reaction time as function of alert-trial number (median across blocks per participant, mean across participants, errorbars omitted for display clarity), **e**) histogram of correlation coefficients for the Spearman correlation between the alert-trial number (1..64) and the percentage of completely correct alert-trials across all blocks in the respective trial for each individual. **f**) histogram of correlation coefficients for the Pearson correlations between the alert-trial number (1..64) and the median reaction time across blocks in the respective trial for each individual.

Supplementary Material, Part 5: Individual Sensitivity

To facilitate comparison of overall performance with other tasks and studies, we characterized the participants' response to alerts by a signal detection theory measure, again aggregating across experiments and conditions. For this analysis, we consider it

- a "hit" if the observer responds to the alert in an alert trial (i.e., gives an up or down response, shows no alert-task intrusion),
- a "correct rejection" if the observer responds to the primary task in a non-alert trial (i.e., gives a left or right response, shows no primary-task intrusion),
- a "miss" if the observer responds to the primary task in an alert trial (i.e., gives a left or right response, shows an alert-task intrusion), and
- a "false alarm" if the observer responds to along the up/down axis despite no alert present in the trial (i.e., shows a primary-task intrusion).

The hit rate (p_{hit}) is usually given as the number of hits divided by the number of alert trials (hits + misses) and the false alarm rate (p_{FA}) by the number of false alarms divided by the number of no-alert trials (false alarms and correct rejections). Since performance in many cases is close to ceiling, we apply the correction formulae[S7]:

$$p_{hit} = (\#hits + 0.5)/(\#hits + \#misses + 1)$$

$$p_{FA} = (\#false\ alarms + 0.5) / (\#false\ alarms + \#correct\ rejections + 1)$$

From these values we compute the sensitivity d' and the criterion c:

$$d' = z(p_{hit}) - z(p_{FA})$$

$$c = -0.5\ (z(p_{hit}) + z(p_{FA}))$$

We find that all participants exhibit d' values above 3.8 (Fig. S5a). In addition, all participants exhibit criteria substantially larger than 0 (Fig. S5b), that is, they apply a conservative criterion. This means, participants are more likely to erroneously respond to the primary task when an alert is present (i.e., to miss the alert) than to erroneously respond to an alert when no alert is present. Interestingly, sensitivity increases with age ($r(77) = .330$, $p = .003$, Fig. S5c) and the criterion decreases (becomes less conservative) with age ($r(77) = -.425$, $p < .001$, Fig. S5d). It should be noted, however, that our sample is comparably age-homogeneous and the experiments were not designed to assess individual differences.

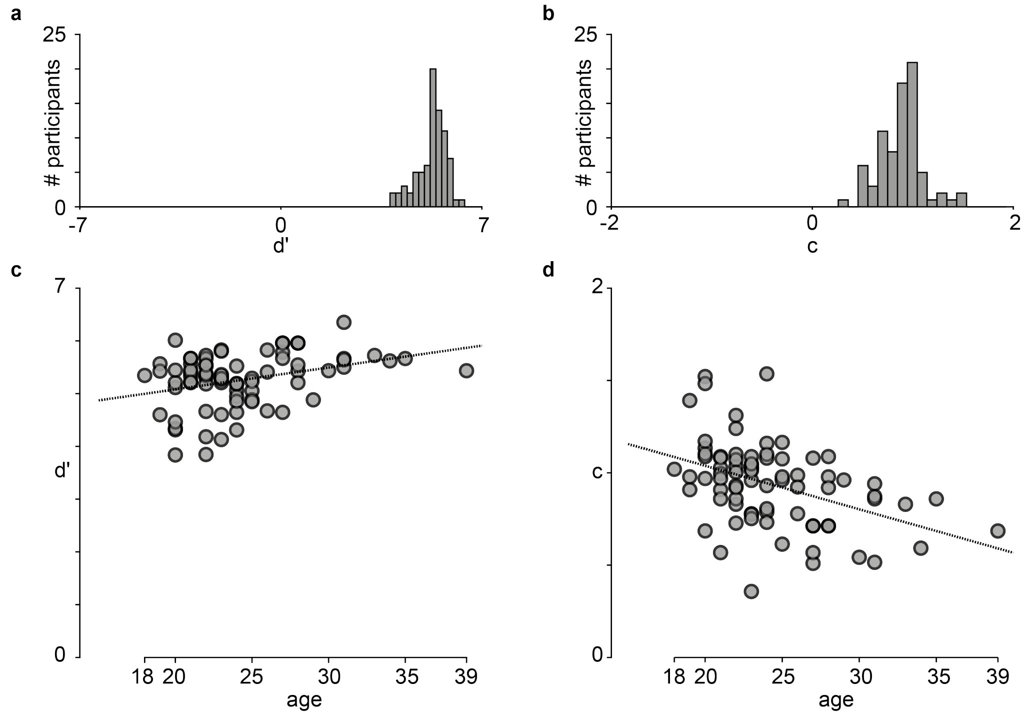

Supplementary Figure S5. Individual sensitivity and criterion. a) Histogram of d' values for the 79 participants across all experiments. **b)** Histogram of criteria applied by the 79 participants. **c)** correlation between age and d', **d)** correlation between age and criterion.

References cited only in Supplementary Material

S1. Itti, L., Koch, C., & Niebur, E. (1998). A model of saliency-based visual attention for rapid scene analysis. *IEEE Transactions on Pattern Analysis and Machine Intelligence*, *20*(11), 1254-1259.

S2. Harel, J., Koch, C., & Perona, P. (2006). Graph-based visual saliency. Advances in neural information processing systems, 19.

S3. Hou, X., Harel, J., & Koch, C. (2012). Image Signature: Highlighting sparse salient regions. *IEEE Transactions on Pattern Analysis and Machine Intelligence, 34*(1), 194–201. https://doi.org/10.1109/TPAMI.2011.146

S4. Schauerte, B., & Stiefelhagen, R. (2012). Predicting human gaze using quaternion DCT image signature saliency and face detection. In *2012 IEEE Workshop on the Applications of Computer Vision (WACV)* (pp. 137-144). IEEE.

S5. Soltani, A., & Koch, C. (2010). Visual saliency computations: mechanisms, constraints, and the effect of feedback. *Journal of Neuroscience*, *30*(38), 12831-12843. https://doi.org/10.1523/JNEUROSCI.1517-10.2010

S6. Stilwell, B. T., & Gaspelin, N. (2021). Attentional suppression of highly salient color singletons. *Journal of Experimental Psychology: Human Perception & Performance, 47*(10), 1313–1328. https://doi.org/10.1037/xhp0000948

S7. Hautus M. (1995). Corrections for extreme proportions and their biasing effects on estimated values of d'. *Behavioral Research Methods, Instruments and Computers, 27*, 46–51.
